# Emotions in Intergroup Contact: Incidental and Integral Emotions' Effects on Interethnic Bias Are Moderated by Emotion Applicability and Subjective Agency

**DOI:** 10.3389/fpsyg.2021.588944

**Published:** 2021-05-28

**Authors:** Stefania Paolini, Jake Harwood, Aleksandra Logatchova, Mark Rubin, Matylda Mackiewicz

**Affiliations:** ^1^School of Psychology, the University of Newcastle, Callaghan, NSW, Australia; ^2^Department of Communication, University of Arizona, Tucson, AZ, United States

**Keywords:** intergroup contact, perceived agency, emotion source, emotion applicability, intergroup bias, incidental emotion, integral emotion, prejudice

## Abstract

This research draws from three distinct lines of research on the link between emotions and intergroup bias as springboard to integrative, new hypotheses. Past research suggests that emotions extrinsic to the outgroup (or “incidental”), and intrinsic to the outgroup (or “integral”), produce valence-congruent effects on intergroup bias when relevant or “applicable” to the outgroup (e.g., incidental/integral anger and ethnic outgroups). These emotions produce valence *in*congruent effects when irrelevant or “non-applicable” to the outgroup (e.g., incidental/integral sadness and happiness, and ethnic outgroups). Internally valid and ecologically sound tests of these contrasting effects are missing; hence we examined them experimentally in meaningful settings of interethnic contact. To this end, we hybridized established research paradigms in mood and intergroup contact research; this approach enabled us to use same materials and induction methods to instigate incidental and integral emotions in a single research design. In Experiment 1, White Australian students (*N* = 93) in *in vivo* real face-to-face contact with an ethnic tutor in their classroom displayed less interethnic bias when incidentally sad (vs. happy) or integrally happy (vs. sad). In Experiment 2, White American males' (*N* = 492) anti-Arab bias displayed divergent effects under incidental vs. integral (non-applicable) sadness/happiness and similar effects under incidental vs. integral (applicable) anger. The role of perceptions of agency in the emotion-inducing situation is also explored, tested, and explained drawing from mainstream emotion theory. As expected, integral and incidental *applicable* emotions caused valence congruent effects, at the opposite sides of the subjective agency spectrum, by encouraging the generalization of dislike from the outgroup contact partner to the outgroup as a whole. On the other hand, incidental-non-applicable emotions caused valence-incongruent effects on bias, under high agency conditions, by encouraging (non-partner-centered) heuristic processing. Because of the improved methodology, these effects can be regarded as genuine and not the byproduct of methodological artifacts. This theory-driven and empirically sound analysis of the interplay between emotion source, emotion applicability and subjective agency in intergroup contact can increase the precision of emotion-based bias reduction strategies by deepening understanding of the emotion conditions that lead to intergroup bias attenuation vs. exacerbation.

## Introduction

Intergroup contact theory's expansive literature on the reduction of outgroup prejudice via intergroup interactions (e.g., Allport, 1954; Pettigrew and Tropp, 2013) includes extensive analyses of intergroup anxiety (Paolini et al., 2016), some data on intergroup empathy (Pettigrew and Tropp, 2008 for a synthesis), and preliminary investigations of discrete emotions (e.g., Seger et al., 2016; Visintin et al., 2017). However, this literature lacks sophistication in its classification of emotions (Hayward et al., 2017). After decades of research, contact theory is unable to predict or explain with precision when and how people rely on their emotions during interactions with outgroup members to infer how they feel about the stigmatized outgroup. This literature hence needs a robust theory-driven framework explicating the conditions under which, and the mechanisms whereby, emotions influence intergroup bias during contact (Kauff et al., 2017; Fuochi et al., 2020).

Our research sought to (1) clarify which emotions during intergroup contact lead to bias attenuation vs. exacerbation, (2) understand the moderators and mediators of these effects, and thus (3) support the design of more effective bias reduction intervention strategies. At the foundation of this work is an understanding of *emotion source* and *emotion applicability*; therefore we start by presenting these concepts. Later we introduce *subjective agency* as another important factor.

### Emotion Source, Emotion Applicability, and Intergroup Bias

*Where* feelings and emotions come from during intergroup contact *matters*. Feelings stemming from outgroup partners' characteristics and behavior are more strongly associated with outgroup prejudice than feelings stemming from other aspects of the contact situation (e.g., contextual features, like the physical properties of the surroundings; Graf et al., 2014). Hence, the *source* of emotions during intergroup contact moderates the emotion-prejudice link.

Bodenhausen (1993) distinguishes between sources of emotions that are *integral* and *incidental to the outgroup* in intergroup experiences. An emotion is *integral* to the outgroup when it stems from and thus is intrinsic to the outgroup partner (and thus the outgroup). A White Australian's anger from interacting with an obnoxious ethnic Australian is an integral emotion. In contrast, emotions are *incidental* to the outgroup when they stem from factors unrelated and extrinsic to the outgroup partner or the outgroup (e.g., the sadness of a White Australian interacting with an ethnic Australian, when the mood emanates from the weather, background music, or the behaviors of another White individual at the scene).

This dichotomy between integral and incidental emotions has distinguished separate research traditions, but has remained underutilized in the contact literature. We will demonstrate that emotion source (Experiment 1), combined with emotions' applicability to the outgroup (Experiment 2) have distinct effects (bias attenuation/exacerbation) on intergroup bias during contact. This will allow us to propose significant conceptual and empirical advancements in intergroup contact theory. We start by discussing prior research on emotion source and applicability.

#### Integral Emotions Produce Valence-Congruent Effects in Contact Research

Intergroup contact research has routinely assessed the biasing consequences of diffuse *integral* feelings. Positive feelings from pleasant intergroup interactions predict positive intergroup judgments; negative feelings from suboptimal interactions predict negative judgments (e.g., more intergroup bias and stereotyping: Kunda et al., 2002; Pettigrew and Tropp, 2008). These valence-congruent effects extend to pointed, discrete integral emotions during contact: Admiration and sympathy for an outgroup partner predict less outgroup bias, whereas anger, disgust, and anxiety about the outgroup partner predict more bias (Seger et al., 2016; Hayward et al., 2017; Kauff et al., 2017). We return to the distinction between diffuse vs. pointed emotions when introducing the idea of variations of subjective agency around emotion experiences in intergroup contact.

These valence-congruent effects of emotions integral to outgroup partners are explained invoking the heuristic (partner-centered) process of member-to-group generalization (Brown and Hewstone, 2005): Emotions during contact become judgments of individual outgroup contact partners (e.g., warmth/liking/bias of individual ethnic Australians), and generalize to judgments of the outgroup as a whole (e.g., warmth/liking/bias against ethnic Australians in general; Smith, 1993; see also Paolini et al., 2006). A direct and complete test of this mediational process is missing (Paolini et al., 2006, 2016; Kauff et al., 2017); it will be a focus of Experiment 2.

#### Incidental Emotions Produce Valence-Incongruent Effects in Early Mood Research

Early mood researchers documented *counterintuitive* valence-*in*congruent consequences on intergroup judgments of positive emotions *incidental* to the outgroup (i.e., stemming from sources other than the outgroup). In this literature, intergroup bias was often *higher*, not lower, under incidental happiness vs. sadness (Isen and Daubman, 1984; Esses and Zanna, 1995).

These effects are explained by variations in (non-partner-centered) heuristic vs. systematic processing: Positive incidental emotions signal safety and thus encourage heuristic and cursory processing of stimuli (i.e., more bias; Bodenhausen et al., 1994a), driven by the sense that the environment is non-threatening. Negative incidental emotions signal potential problems for the organism, and thus enhance attention and vigilance for threat cues, thus instigating systematic processing that leads to less category-based judgments (Huntsinger et al., 2010) and consequently reduced bias (Brown and Hewstone, 2005; however cf. Amodio and Devine, 2006). Our research will check these accounts by measuring these non-partner-centered putative processes.

#### Incidental Emotions' Effects Are Moderated by the Applicability of Emotions to the Outgroup

This seemingly neat separation between valence-congruent (vs. incongruent) effects of integral (vs. incidental) emotions has been challenged by research on *emotion applicability*. DeSteno et al. (2004); Dasgupta et al. (2009) show that negative incidental emotions “behave” like negative integral emotions and *increase* intergroup bias in valence-congruent (vs. counterintuitive) ways *when* these emotions are perceived to be *applicable*, relevant to the outgroup, or stereotypically associated with the outgroup. For instance, Dasgupta et al. (2009) incidentally induced participants' anger or disgust (negative emotions stereotypically linked with and chronically instigated by Arabs and homosexuals, respectively: Shaheen, 2003; Hodson and Costello, 2007). Incidental anger increased bias against Arabs (relative to a neutral incidental emotion control condition), but incidental disgust did not. Likewise, incidental disgust exacerbated bias against homosexuals, but incidental anger did not.

Hence, for a White Australian, anger stemming from irritating music in the background of an interaction with an ethnic Australian would exacerbate interethnic bias, similarly to anger stemming from interacting with an obnoxious ethnic Australian. This would occur because—irrespective of its source—anger is experienced as being *applicable* to non-White, ethnic Australians (Augoustinos et al., 1994; Stephenson, 2009), as it is to competitive interethnic relations in many Western societies (Cottrell and Neuberg, 2005). When anger (vs. sadness) is experienced by Whites during interethnic interactions, DeSteno and Dasgupta believe that this emotion should facilitate the retrieval of outgroup-stereotype information and behaviors from memory. This schematic knowledge drives the intergroup judgment, resulting in increased bias. Consistent with this view, intolerant intergroup ideologies mediate the link between individuals' chronic disgust sensitivity and homophobia (e.g., Hodson and Costello, 2007). Evidence that outgroup schemas mediate episodic effects of applicable incidental emotions is however unavailable [Dasgupta et al., 2009, p. 589; however see indirect evidence by Fuochi et al. (2020)]; we will test this mediation path.

The evidence that *negative incidental-applicable emotions* “behave like” negative emotions that are integral to the outgroup is noteworthy, as it blurs the traditional view that incidental and integral emotions are fundamentally different and have different downstream consequences. Our research will demonstrate that emotion applicability significantly influences the emotion-prejudice link in intergroup contact by moderating the effects of emotion source in ways that are consistent but more nuanced than Dasgupta and DeSteno's original effects as detected away from realistic contact settings.

### Integrating and Contrasting Distinct Emotions' Effects

Our understanding of emotions in intergroup contact is currently limited because the three lines of research we sketched above have focused on *either* incidental *or* integral emotion sources; as a result, they have used largely incommensurable methodologies (different emotion induction methods, designs, and controls). These methodological discontinuities, and the lack of single-design studies encompassing incidental *and* integral emotions, prevent comparison of emotion effects across lines of research and the identification of (shared vs. unique) mechanisms responsible for the emotion-prejudice link (Mackie et al., 1996; Wilder and Simon, 1996; Bodenhausen et al., 2001).

**Figure 1:**
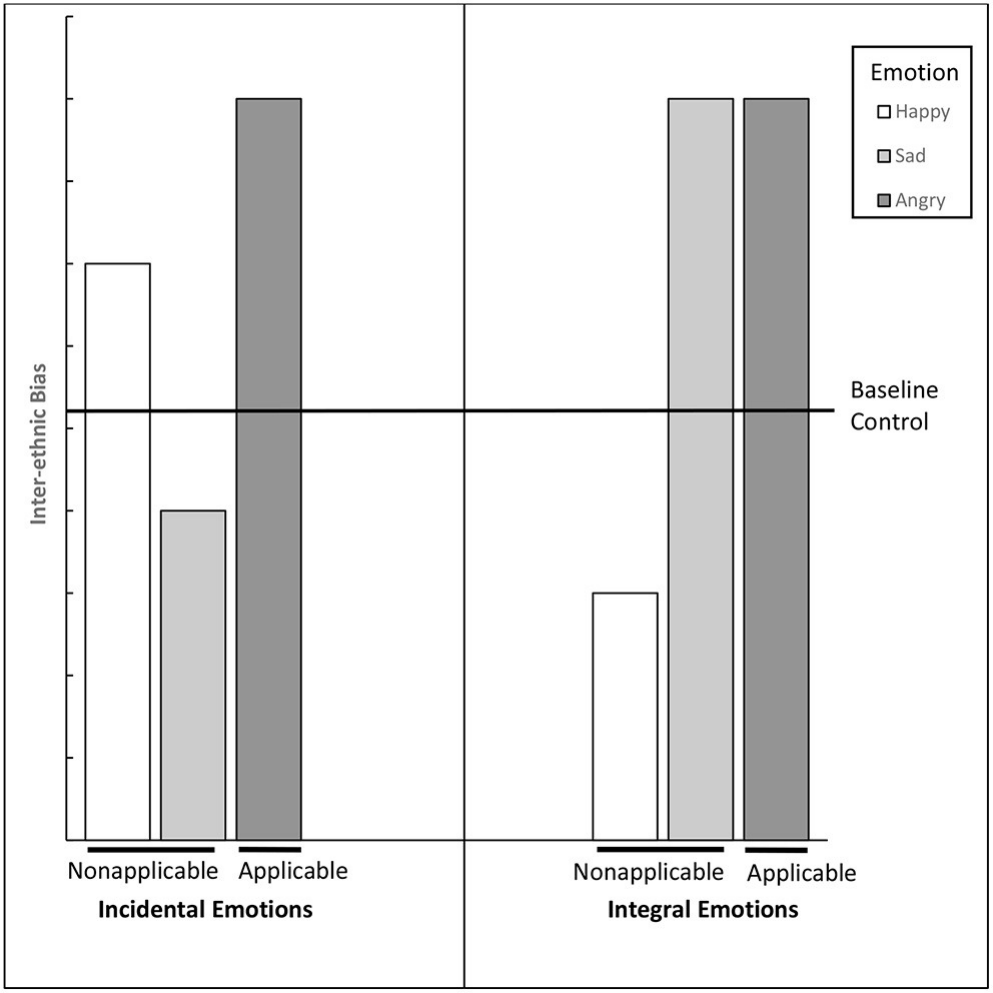
Pattern of *predicted* effects of emotions on interethnic bias as a function of emotion source (incidental = extrinsic to the outgroup partner; integral = intrinsic to the outgroup partner), and emotion applicability (applicable = relevant to the outgroup; non-applicable = irrelevant to the outgroup), as extrapolated from three independent lines of past emotion research. The inferred pattern suggests the existence of two broad emotion effects on bias: (1) opposite effects for incidental/integral-non-applicable emotions (cf. incidental/integral happy bars vs. baseline and incidental/integral sad bars vs. baseline; *Prediction 1*); (2) similar effects for incidental/integral-applicable emotions (cf. incidental/integral angry bars vs. integral happy/baseline; *Prediction 2*).

Figure 1 brings together the three lines of research discussed so far, contextualizing their findings with reference to competitive *interethnic* relations. In the left panel, the contrast between the first two bars displays the counterintuitive effects of positive and negative non-applicable *incidental* emotions (from early emotion research), while the contrast between the second and third bars shows the emotion applicability effect for negative incidental emotions (per DeSteno-Dasgupta). The right panel shows the valence-congruent effects of positive and negative integral emotions reported in the contact literature: positive emotions in contact reduce bias and negative emotions in contact increase bias. Our synthesis of otherwise disjoint traditions helps us re-formulate earlier findings in terms of two broader and untested predictions about the biasing effects of emotions in interethnic contact settings.

#### Predictions for the Emotion-Prejudice Link as a Function of Source and Applicability

##### (1) Opposite Effects of Incidental-Non-Applicable vs. Integral-Non-Applicable Emotions

Emotions that are incidental and integral to the outgroup will have divergent effects on interethnic bias when non-applicable to the outgroup: Incidental-non-applicable emotions will have valence-*in*congruent effects, whereas integral-non-applicable emotions will have valence-congruent effects. In interethnic settings, for instance incidental happiness will cause more bias than incidental sadness, but integral happiness will cause less bias than integral sadness.

##### (2) Similar Effects of Incidental-Applicable and Integral-Applicable Emotions

Negative emotions that are incidental and integral to the outgroup will have convergent effects on interethnic bias when applicable to the outgroup: Both incidental-applicable and integral-applicable emotions will have valence-congruent effects. Hence, incidental and integral anger should *both* lead to more bias.

Confidence in the existence of these effects is hindered by the aforementioned methodological discontinuities across lines of enquiry (Mackie et al., 1996; Wilder and Simon, 1996; Bodenhausen et al., 2001). For example, Figure 1 extrapolates bias levels for baseline control (see continuous horizontal line). Past studies varied in whether they included control conditions (e.g., contact research often did not) and in the type of control they used (e.g., no emotion induction, emotion neutrality). As a result, some of this research does not differentiate simple bias levels from bias attenuation/exacerbation (relative to baseline). Such methodological discontinuities inject uncertainty into our integrative predictions.

To our knowledge, there are only two attempts at explicitly contrasting incidental and integral emotions' effects on intergroup judgments. In separate studies, Wilder and Shapiro (1989a,b) induced *incidental* anxiety (i.e., anxiety about a dentist procedure) and *integral* anxiety (i.e., anxiety around intergroup competition). Consistent with Prediction 2, they found that negative incidental-applicable and negative integral-applicable emotions had similar, valence-congruent biasing effects on intergroup judgments: anxiety increased bias irrespective of source type. Echebarria and Fernandez (2004) used a mixture of emotion induction methods to incidentally and integrally induce anger, sadness, and neutrality in a single study of competitive intergroup relations between student groups. Contrary to DeSteno et al. (2004) and Dasgupta et al. (2009), they found no reliable difference in bias between negative incidental-applicable and non-applicable emotions and a complex and inconsistent pattern for integral emotions. Because of methodological discontinuities, only tentative comparisons can be made between these studies and our predictions (Mackie et al., 1996; Wilder and Simon, 1996).

### This Research's Aims and Methods

Our primary aim was to provide the first *un*confounded tests of the novel, overarching Predictions 1 and 2 for the emotion-prejudice link in interethnic contact settings. We carried out these tests looking at interethnic relationships in Australia and the US. In Experiment 1 we focused on Prediction 1, as key to demonstrate that emotion source can produce marked dissociations in bias and thus needs considering in intergroup settings. We induced happiness and sadness (positive and negative emotions non-applicable to competitive ethnic outgroups: Fiske, 2002), while systematically varying emotion source (incidental vs. integral). In Experiment 2, we manipulated both emotion applicability and source to test both Predictions 1 and 2. Again, we induced happiness and sadness and manipulated emotion source (incidental vs. integral). We added anger, a negative emotion relevant (applicable) to the focal ethnic outgroup (Augoustinos et al., 1994; Stephenson, 2009). By hybridizing past methods within a single design, and using the same materials to systematically vary emotion source (Experiments 1 and 2) and emotion applicability (Experiment 2), we were able to rule out methodological confounds and thus improve on earlier findings' internal validity.

In addition, we endeavored to move tests of emotions incidental to the outgroup to more realistic settings of interethnic contact. We focused on interethnic relationships in intergroup contact settings because of their social relevance and psychological significance in Australia and the US (Shaheen, 2003; Soutphommasane, 2012). This approach enhanced ecological validity, and provided ideal conditions to explore novel mechanisms (moderators/mediators) specific to emotions occurring in multifaceted realistic settings (vs. the laboratory).

Finally, this research offered a fresh and serendipitous opportunity to bring emotion appraisal theory straight within the realm of intergroup contact research. In Experiment 1, encouraged by an anonymous reviewer, we provide an analysis of the emotion source effects on bias in terms of subjective agency of the emotion source and contact partner. In Experiment 2 we introduce prospectively moderation tests for subjective agency around the emotion source and the contact partners. This factor is novel to the contact literature, but well-established in mainstream emotion and attribution research. We see it as adding an important and generative new layer of analysis of contact dynamics.

Ultimately, we draw from nuanced considerations of emotion source, emotion applicability, and subjective agency to explore whether individuals use emotions in contact to infer how they feel toward outgroups.

## Experiment 1

In Australia, a modern, relatively peaceful democratic society, negative responses to non-Whites and immigrants, and broader anti-multiculturalism sentiments are common in segments of the community, political establishment, and the media (Aslan, 2009; Soutphommasane, 2012). In this climate, we experimentally examined bias toward non-White, ethnic people in our first test of Prediction 1: opposite effects on ethnic bias of positive and negative incidental/integral emotions *non-applicable* to the outgroup.

White Australian students in face-to-face, *in vivo* contact with an unfamiliar Pakistani classroom tutor were induced to feel either happy *or* sad (*emotion* factor) due to a source that was incidental *or* integral to the outgroup partner (*source* factor), prior to completing a measure of interethnic bias and several potential mediators^1^. This tests Prediction 1 on the effects of non-applicable emotions because happiness, positive feelings and emotions, while normative across most settings (Diener and Diener, 1996) and common in interethnic interactions (Graf et al., 2014), are *not* experienced as stereotypically belonging to ethnic outgroups (Fiske, 2002; Mackie et al., 2009), including Pakistanis in Australia (Augoustinos et al., 1994). Similarly, sadness is not stereotypically linked to interethnic relationships in Australia (Aly and Green, 2010).

Drawing on past research (Figure 1), we expected a significant, disordinal emotion by source interaction, featuring opposite effects of incidental/integral-non-applicable emotions. Interethnic bias was expected to be higher in the incidental/non-applicable-happy (vs. sad) condition (a valence-*in*congruent effect), but to be higher in the integral/non-applicable-sad (vs. happy) condition (a valence-congruent effect). By returning a marked dissociation in bias as a function of emotion source for emotions that are non-applicable to the outgroup, this experiment would reinforce a view that an appreciation of emotion source is important in intergroup contact settings.

## Method

### Participants and Design

All students enrolled in a second-year psychology course at a large regional Australian university participated in the research as part of the course's tutorial activities. Ninety-three students (29 male and 64 female; mean age of 22.76 years, *SD* = 6.57) entered the analyses^2^. They all had an Anglo background and were native English speakers. A 2 (emotion source: incidental/integral) × 2 (emotion: happy/sad) quasi-experimental between-subjects factorial design was used: We randomly allocated participants to levels of the emotion source factor, and laboratory classes to levels of the emotion factor (there were 22–24 participants and three classes per condition).

### Procedure, Materials, and Measures

Participants took part in two allegedly unrelated studies during their regular tutorial class for which a young, female (visibly non-Anglo) Pakistani acted as tutor, as part of a course on psychology research methods.

#### Manipulations and Emotion Checks

The *emotion* factor was manipulated by randomly assigning classes to one of two 10-min (ethnicity-unrelated) video clips, as part of a pilot study ostensibly aimed at preparing experimental materials for future research on the evocative power of visual stimuli (Isen and Daubman, 1984). *Happy* participants viewed an excerpt of a popular comedy show and *sad* participants viewed an excerpt of a documentary on illness. We manipulated *emotion source* just prior to showing the emotion-inducing video by varying a message to individual participants about the video assignment process (see Rydell et al., 2008; Mackie et al., 2009, Study 1 for examples of framing of emotion source manipulations). On the cover page of the research booklet, participants learnt that there were two videos available for the research and their video clip had been assigned to their class either “on a random basis (a ticket was drawn out of a hat)” (*incidental* condition) or specifically to them “by your tutor at the very beginning of this class” (*integral* condition). Immediately after watching the video clip, participants completed Tamir and Robinson's (2004) mood scale, including ratings of self-reported happiness and sadness (1 = *not at all*, 7 = *extremely*); these ratings were a manipulation check for the emotion manipulation.

#### Contact Partner's Interview and Emotion Source Checks

Next, as part of a second (ostensibly unrelated) study (Forgas, 1989) on first impressions, participants learnt about their (otherwise unfamiliar) ethnic tutor through a 10-min face-to-face interview in front of the class. A student volunteered to play the role of the interviewer; the tutor was trained to behave neutrally and consistently across conditions while responding to a predefined set of questions. The interview was scripted and based on earlier intergroup contact research (Paolini et al., 2010, Study 1); it included information about the tutor's Pakistani family background, as well as ethnicity-unrelated interests and experiences that a student focus group had identified as representative of students at that university. Next, participants were asked to “take a break” from the second study, completing a “follow-up questionnaire” for the first study. This research booklet measured potential mediators (see Supplementary Material), and included a check for the source manipulation which asked participants to recognize the method used to allocate the video (random vs. choice made by their tutor). All but four participants attended to this critical piece of information^3^.

#### Interethnic Bias

Next, participants completed a measure of interethnic bias embedded among several filler items for non-focal categories (e.g., teachers, women) to limit suspicions about interests in ethnicity. Participants indicated their group-level attitudes or warmth toward ethnic minorities in general (locally labeled as “non-Anglo people”) using a single-item feeling thermometer widely used and validated in intergroup contact research (0 = *extremely unfavorable/cold*, 100 = *extremely favorable/warm*; Haddock et al., 1993). We reverse-scored this measure so that high values indicated more ethnic prejudice or outgroup bias. An end-of-session feedback questionnaire allowed participants to express their thoughts and suspicions about the study; full written and oral debriefing also followed. This and the next experiment's protocol were approved by the institution's human subjects ethics board.

## Results and Discussion

### Emotion Checks

We checked our emotion manipulation with a 2 (emotion source: incidental/integral) × 2 (emotion: happy/sad) between-subjects ANOVA on self-reported happiness and sadness. As expected, on both measures we detected main effects of emotion, both *p*s < 0.001. Participants who were induced to feel happy reported significantly more happiness (*M* = 5.07, *SD* = 0.99) and less sadness (*M* = 1.47, *SD* = 1.12) than participants induced to feel sad (happiness: *M* = 3.46, *SD* = 1.29; sadness: *M* = 3.29, *SD* = 1.70). The ANOVAs detected no effect of emotion source either on its own, or in combination with the emotion factor on the two self-reported emotion indices (all *p*s > 0.24), demonstrating that emotion source had no bearing on these self-reported emotions (Rydell et al., 2008).

### Emotion Effects on Interethnic Bias

**Figure 2:**
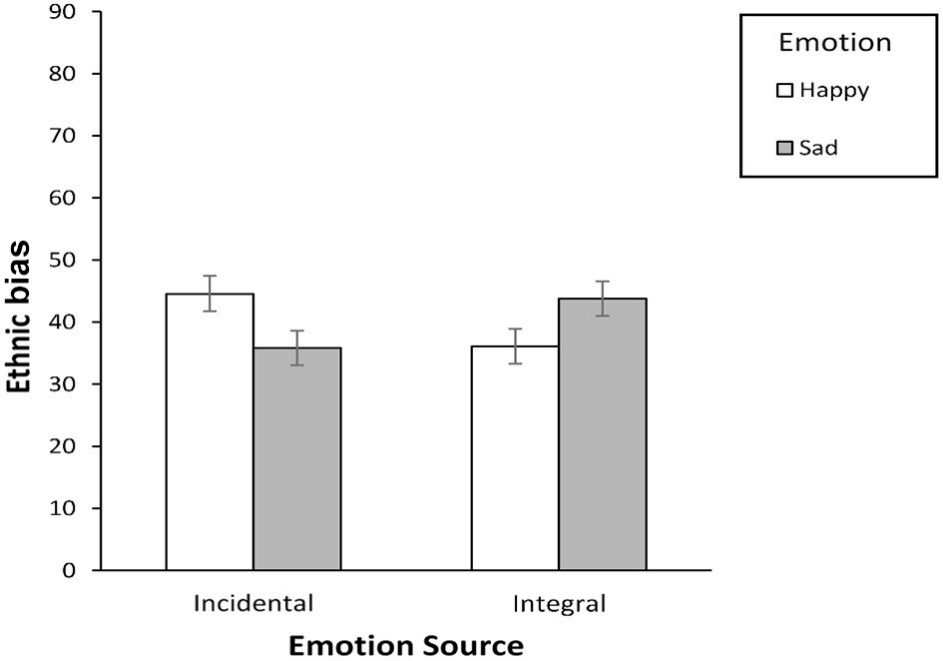
Divergent pattern of effects obtained for positive and negative incidental-non-applicable and integral-non-applicable emotions on interethnic bias consistent with Prediction 1 (Experiment 1; *N* = 93 White Australian students in face-to-face contact with ethnic tutor in their classroom). Graph includes error bars as SEs.

Based on Prediction 1, we expected the ethnic bias index to display opposite effects of incidental- non-applicable and integral-non-applicable emotions in the shape of a disordinal emotion by emotion source interaction. Using G^*^Power 3.1 (Faul et al., 2007), we determined that Experiment 1 had power of 0.80 to detect effect sizes of η^2^ = 0.08, which is slightly larger than typical effects in the field of psychology (Cafri et al., 2010). Hence, this study is slightly underpowered and thus should be replicated in new settings, perhaps with a different outgroup. Nonetheless, our 2 (emotion source: incidental/integral) × 2 (emotion: happy/sad) between-subjects ANOVA on the ethnic bias index yielded a significant interaction effect, *F*_(1, 89)_ = 8.36, *p* = 0.005, η^2^ = 0.09 (see Figure 2; all other *F*s < 1). As predicted, ethnic prejudice was significantly higher among incidental/non-applicable-happy (*M* = 44.55, *SD* = 12.14) than incidental/non-applicable-sad participants (*M* = 35.83, *SD* = 14.72), *F*_(1, 44)_ = 4.72, *p* = 0.035, η^2^ = 0.10, in line with a valence-incongruent effect. Ethnic bias was also marginally lower among integral/non-applicable-happy (*M* = 36.09, *SD* = 16.16) than integral/non-applicable-sad participants (*M* = 43.78, *SD* = 10.95), *F*_(1, 45)_ = 3.68, *p* = 0.061, η^2^ = 0.08, in line with a valence-congruent effect^4^. When decomposing the interaction the other way, a difference in bias between incidental and integral participants was detected in both the non-applicable-happiness conditions, *F*_(1, 43)_ = 3.89, *p* = 0.055, η^2^ = 0.08, and the non-applicable-sadness conditions, *F*_(1, 46)_ = 4.51, *p* = 0.039, η^2^ = 0.09.

Experiment 1 provides the first neat, direct, and unconfounded evidence of the opposite effects of incidental-non-applicable and integral-non-applicable emotions, consistent with Prediction 1. As expected (Figure 1), incidental-non-applicable emotions led to valence-incongruent effects, whereas integral-non-applicable emotions led to valence-congruent effects. Using a single design and emotion induction method, this study unequivocally demonstrates that the distinct patterns of effects we predicted from past mood and contact research (cf. Figure 1's happy-sad incidental vs. integral emotions) reflect genuine, distinguishable emotion effects and not simply methodological discontinuities between research traditions. We found *no* mediational evidence in this experiment to explain the opposing pattern (see Supplementary Material). Explaining our focal effects was therefore one goal of Experiment 2.

### Exploring the Role of Agency

Our method for manipulating emotion source in Experiment 1's naturalistic contact setting was innovative, but not problem-free: As an anonymous reviewer of this study noted, besides varying appraisals of emotions as internal vs. external to the ethnic contact partner as intended, our experimental manipulation of emotion source also potentially varied the perceived agency of the ethnic contact partner. In one condition (incidental: ticket out of a hat) she had no control and thus was low agency. In the other (integral: ethnic partner's decision), she explicitly directed the emotions induced by the treatment, and hence had high agency.

**Figure 3:**
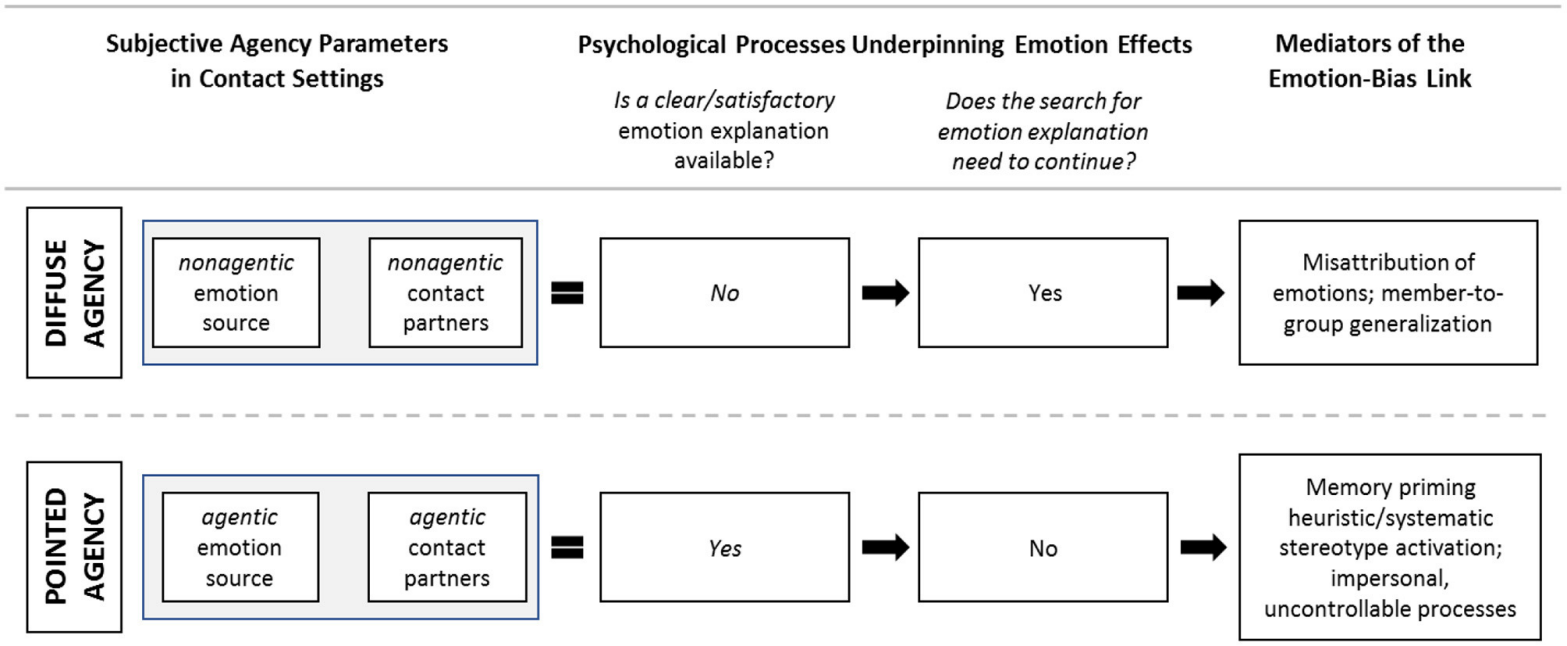
Expectations derived from contemporary emotion appraisal theory and emotion research that variations in the perceived agency of emotion source and contact partners together will impact the interpretation of emotions during contact, affect the processes underpinning judgment construction and thus shape the mediators of the emotion-bias link.

The agency ascribed to the emotion source and contact partners might shape emotion interpretations during contact (Graf et al., 2014; Paolini and McIntyre, 2019). Drawing from contemporary emotion appraisal theory and emotion research (Ellsworth and Scherer, 2003; Scherer and Moors, 2019), we imagine different processes unfolding in situations of diffuse (low) vs. pointed (high) agency. These broad expectations for the downstream consequences of subjective agency in contact are summarized in Figure 3.

Diffuse or low agency situations (see top half of Figure 3) are those in which the emotion source is subjectively difficult to identify or isolate: The emotion source lacks intentionality, and the contact partners are improbable causes due to their low perceived agency. Under such conditions, people actively search for an explanation for their emotions and struggle to find one. One result of this search may be an unconscious misattribution of the emotions to the outgroup member: if I don't know why I'm angry, perhaps it's because of the outgroup member. These misattributions can extend to perceptions of the entire outgroup via member-to-group generalizations commonly found in the contact literature (e.g., Fuochi et al., 2020). Such scenarios of diffuse agency echo the expansive literature on frustration aggression and scapegoating (Dollard et al., 1939; Anderson et al., 2000), as well as research on diffuse, nonsocial emotion sources such hormones or weather (Schwarz and Clore, 1983; Macrae et al., 2002).

Pointed of high agency situations, on the other hand (see bottom half of Figure 3), are those in which the emotion source is clearly identified, and the contact partners are perceived as agentic. In such settings, a clear explanation for the individual's emotion is present (e.g., when a fellow ingroup member is the identified cause of the emotions). Contemporary emotion research tells us that under these conditions, the search for an explanation for one's emotions stops, and incidental emotion effects that are unintentional, uncontrolled and impersonal in nature ensue (Leyens et al., 1992; Forgas, 1994; Ranganath and Nosek, 2008). These effects might take the form of the previously discussed, non-partner-centered heuristic-processing-driven pattern of higher bias with positive emotions. Or they might be valence-congruent effects reflecting memory priming (Bower, 1991) or active stereotypes (Dasgupta et al., 2009). When the *out*group member is the identified cause of such emotion and the participant attributes responsibility for their emotions to their (agentic) contact partners, intergroup processes of outgroup member-to-group generalization will be at their strongest, as the outgroup member is “held directly responsible” for the emotions (whether positive or negative; Graf et al., 2014).

To explore these ideas, Experiment 2 systematically manipulates the agency of the emotion source (low/high) and assesses variations in the agency of the contact partners (low/high). In so doing, this study orthogonally varies parameters that we expect to drive subjective experience of agency in contact settings and associated emotion appraisals (see Figure 3's left-hand side); it teases apart features of the emotion experience that in Experiment 1 overlapped with our emotion source manipulation thus helping us delve into implicated processes.

To directly check the effects of *emotion source* independently of agency, our enriched design features an ingroup member as an emotion source incidental to the outgroup who is just as agentic as the outgroup member used to operationalize the integral emotion source, meaning differences between these two conditions can only be a function of source (not agency). Therefore, some participants' emotions stemmed from an (agentic) *out*group member (*integral/outgroup* condition) while other participants' emotions came from an (equally agentic) *in*group member (*incidental/ingroup* condition).

To directly check the effects of emotion *source agency* per se, we included in our design also a *non-agentic* incidental source condition—for these participants, emotions stemmed from environmental background music (*incidental/music* condition). Having both an incidental/ingroup and an incidental/music condition thus clarifies whether the agentic vs. non-agentic, as well as the social vs. non-social nature of incidental sources matter.

Finally, to gage natural variation in perceptions of contact partners' agency and directly check the effects of this factor on the emotion-prejudice link, we gathered overall perceptions of the contact partners' ability to make autonomous decisions. We aimed for this measure to capture participants' general willingness to grant others responsibility over their contact experience and emotions during contact.

Several of the mediators contemplated in Figure 3 (see figure's right hand) were also included. Experiment 2 thus tested concepts of subjective agency (causation/determination) that have received some attention in emotion and attribution research (Ellsworth and Scherer, 2003; Moors et al., 2013) but are novel in contact research. We examine whether subjective agency shapes the emotion-prejudice link during interethnic contact.

## Experiment 2

Experiment 2 furthered our understanding of emotion-prejudice effects, ascertaining the internal validity of past emotion findings as displayed in Figure 1, and isolating possible mechanisms (moderators/mediators) of these effects in interethnic contact settings. With an extended design and again a single emotion induction method, this time we tested both our predictions, that (1) incidental/integral-non-applicable emotions would have *opposite* (valence-incongruent/congruent) effects on interethnic bias (Prediction 1), and that (2) incidental/integral-applicable emotions would have *similar* (valence-congruent) effects on bias (Prediction 2). We achieved this by orthogonally manipulating *emotion* and *emotion source* within an imagined contact paradigm (Husnu and Crisp, 2010), which could better handle the large fully-powered between-subjects design we needed, and which afforded plenty of variability in subjective agency. Growing evidence suggests that imagined contact produces effects, and harnesses mechanisms that parallel real intergroup contact (Miles and Crisp, 2014). We return to the ecological validity of these methods in the general discussion.

White male Americans visualized interacting with two unfamiliar individuals: another White American (ingroup member) and an Arab American (outgroup member), introduced exclusively via two photographs and typical European/Arab names. Based on condition, participants imagined feeling *happy* (positive/non-applicable emotion), *sad* (negative/non-applicable emotion), or *angry* (negative/applicable emotion) as a result of either music played in the background (non-agentic *incidental/music* source) or as a result of what was said or done by either the White contact partner (agentic *incidental/ingroup* source) or the Arab contact partner (agentic *integral/outgroup* source). An appended control group was also included who engaged in a filler visualization task, thus providing a baseline. All participants reported on their anti-Arab bias following their treatments.

We expected a significant *two-way* emotion by emotion source interaction to emerge on interethnic bias. Finding divergent effects on bias of happy/sad incidental vs. happy/sad integral participants would replicate Experiment 1's results and confirm Prediction 1. Finding convergent effects on bias of angry/incidental and angry/integral participants would provide novel evidence for Prediction 2.

Based on the discussion of agency above, we explored the possibility of a higher-order interaction driven by variations in source and contact partner agency. Hence, we included an exploratory measure of contact partners' perceived agency as an additional between-subjects factor in the analyses, and predicted a significant emotion by emotion source by partner agency interaction on interethnic bias.

Moreover, we broadened and refined our tests of mediators (cf. Supplementary Material for Experiment 1 and Footnote 1): We introduced measures of bias/dislike of the Arab American contact partner (and for completeness of the White American contact partner and the ingroup) to test for member-to-group generalization. We also used new, more direct indices of stereotype activation and non-partner-centered heuristic processing. We expected outgroup member-to-group generalization to mediate incidental emotion effects on bias in the non-agentic incidental/music condition under low contact partner agency (i.e., the most diffuse agency end; Figure 3 top). However, we expected stereotype activation and heuristic processing to mediate incidental emotion effects on bias in the agentic incidental/ingroup condition under high partners agency (i.e., the most pointed agency end; Figure 3 bottom). If corroborated, these moderation effects and conditional mediational findings would confirm that subjective agency during contact affects whether and how emotions are used in these settings to infer feelings toward the outgroup (Graf et al., 2014).

## Method

### Participants and Design

Self-identified US-born, native English-speaking male adults of Caucasian background (*N* = 492; 26.4% 18–25 years; 54.5% 26–35 years, 19.1% 36–45 years) were recruited on Amazon's MTurk crowdsourcing platform with a monetary incentive (US$2 plus $0.06 for the eligibility screening). They consented to their data inclusion after full written debriefing. Using G^*^Power 3.1 (Faul et al., 2007), we determined that Experiment 2 had power of 0.80 to detect effect sizes of η^2^ = 0.02, which is considerably smaller than typical effects in the field of psychology (Cafri et al., 2010) and smaller than the typical imagined contact effect reported in a meta-analysis (Miles and Crisp, 2014); hence, this experiment was adequately powered. The study used a 3 (emotion: happy-non-applicable, sad-non-applicable, angry-applicable) × 3 (source: incidental/music, incidental/ingroup, integral/outgroup) fully factorial between-subjects design with an appended no-visualization control. Participants were assigned randomly to conditions (*n* = 44-62 per condition).

### Procedure

#### Visualization Scenarios

A “visualization study” was made available online to prospective participants who indicated on a demographic filter that they were male, 18-45 years old, English-native speakers, White, and US-born. We focused on males because some literature suggests that intergroup competition in interethnic settings is primarily a male affair (McDonald et al., 2012; Vandello and Bosson, 2013).

Those randomly assigned to an experimental condition were presented with two photographs: one of a Caucasian-looking man, allegedly called “Matthew,” and one of an Arab-looking man, “Haashim” (for name sourcing, see Park et al., 2007), randomly drawn from three White-Arab photograph pairs to minimize stimuli sampling biases (Judd et al., 2012). The provision of an ingroup and an outgroup target ensured that the intergroup categorization was equally salient across experimental conditions (Brown and Hewstone, 2005) and provided a common basis for the intergroup visualization. Participants were asked to imagine attending a friend's party, where they approached the two unfamiliar men talking to each other. Depending on condition, participants visualized feeling either *happy, sad*, or *angry* (*emotion* manipulation) as a result of music playing in the background (*incidental/music* source) or something that Matthew (*incidental/ingroup* source) or Haashim (*integral/outgroup* source) did or said. Participants freely described the content of their visualization in response to two open-ended questions prompting elaboration (Husnu and Crisp, 2010). Control participants visualized walking through an outdoor scene of their choice (see Miles and Crisp, 2014) and also freely described the content of their visualization.

#### Manipulation and Attention Checks

To check the effectiveness of the emotion manipulation, participants indicated the degree to which they felt *happy, sad* and *angry* among other emotions (Tamir and Robinson, 2004; 1 = *not at all*, 9 = *extremely*). To check engagement, we included a general attention check (Paolacci et al., 2010), ethnicity checks for the characters in the story (with multiple options, valid answers = White/Arab for Matthew/Haashim, respectively); an emotion check on which respondents recalled the emotion they had been asked to focus on during the imaginary interaction, and a check on recall for the source of that emotion. Eighty-five per cent of the participants (*N* = 492 of 582) passed all the attention checks and were retained for the analyses.

#### Key Measures

To disguise our aims and avert anchoring effects, key measures were embedded among fillers. *Bias* toward the outgroup contact partner (Haashim), the outgroup as a whole (Arabs), the ingroup contact partner (Matthew), and the ingroup as a whole (Whites) were assessed using feeling thermometers (Haddock et al., 1993; see Experiment 1: 0° = *Very cold/unfavorable*, 100° = *Very warm/favorable* with 10° increments). We reverse scored these indices, so that high values reflected more *bias* (dislike of contact partners/groups). To explore perceived agency of the contact partners, participants indicated the extent to which “active” and “makes decisions easily” described Haashim and Matthew (derived from Alkire, 2005; see also “activity” and “potency” in Osgood et al., 1957; 1 = not at all, 9 = very much; 4 items, alpha = 0.79). Our manipulations did not affect this contact partner agency index, consistent with its planned moderating role, all *ps* > 0.32. We prepared this index to be our third between-subject factor (low vs. high contact partner agency; *Mdn* = 5.75 on a 1-9 scale)^5^.

Heuristic processing was measured with three items. Participants indicated the extent to which “during the visualization…. [they] didn't spend much time thinking about what [they were] asked to do”; “they stopped and thought carefully about the task” (R) and “thought about each person carefully (i.e., Matthew and Haashim)” (R; 0 = *not at all*, 9—*very much*). A reliable heuristic index was computed (Cronbach's alpha = 0.73); high values indicated more non-partner-centered heuristic processing.

We used an Implicit Association Test (or IAT) modeled on Greenwald et al. (2003) to assess implicit stereotype activation of the Arab-barbaric and White-greedy stereotypes (Oswald, 2005; Conley et al., 2010). In the critical blocks, participants sorted “as fast and as accurately as possible” individual barbaric and greedy words (e.g., fundamentalist, brutal vs. selfish, privileged) between compatible (i.e., Arab-White) and incompatible (i.e., White-Arab) categories. To control for evaluative responding (Amodio and Devine, 2006), all target words were negative; the compatible-incompatible blocks were counterbalanced across participants. *Implicit stereotype activation* was indexed in terms of relative differences in latencies between sorting compatible vs. incompatible word pairings following Greenwald et al.'s screening method. Positive D-indices indicated faster responses for White-greedy *and* Arab-barbaric pairings, relative to White-barbaric and Arab-greedy pairings, thus signaling implicit activation of *both* groups' *stereotypes*. Control participants did not rate the contact partners, but provided only group-level bias measures to allow us to establish where bias exacerbation vs. attenuation occurred as a result of the manipulations.

The implicit measure of stereotype activation confirmed that overall participants endorsed the in/outgroup stereotypes (Arabs-barbaric; White-greedy), D-index *M* = 0.41, *SD* = 0.38, one-sample *t*_(491)_ = 23.72, *p* < 0.001. Moreover, experimental participants reported a significantly higher D-Index, *M* = 0.43, *SD* = 0.37, than the control participants, *M* = 0.28, *SD* = 0.38; *F*_(1, 473)_ = 7.05, *p* = 0.008, $\eta_{p}^{2}$ = 0.015, confirming that the intergroup visualization had boosted stereotype activation. All participants were debriefed.

## Results and Discussion

### Emotion Checks

We checked the effectiveness of our emotion manipulations with 3 (emotion: happy/sad/angry) × 3 (emotion source: music/ingroup/outgroup) between-subjects ANOVAs on self-reported happiness, sadness and anger. As expected, we found only a significant main effect of emotion on self-reported happiness, *F*_(2, 421)_ = 26.36, *p* < 0.001, $\eta_{p}^{2}$ = 0.111, and sadness, *F*_(2, 421)_ = 43.82, *p* < 0.001, $\eta_{p}^{2}$= 0.172, all other effects *ps* > 0.05. Those who visualized feeling happy reported more happiness (*M* = 6.02, *SD* = 2.09) than those who visualized feeling sad (*M* = 4.26, *SD* = 2.15) or angry (*M* = 4.75, *SD* = 2.14), both *p*s < 0.001 (sad-angry difference *ns*). Participants who visualized feeling sad reported more sadness (*M* = 4.03, *SD* = 2.54), than those who visualized feeling angry (*M* = 2.68, *SD* = 2.02) or happy (*M* = 1.80, *SD* = 1.44); the angry-happy difference was also significant, all *ps* < 0.001. Hence, the happy and sad manipulations elicited the desired emotions and their reporting was unaffected by emotion source.

**Figure 4:**
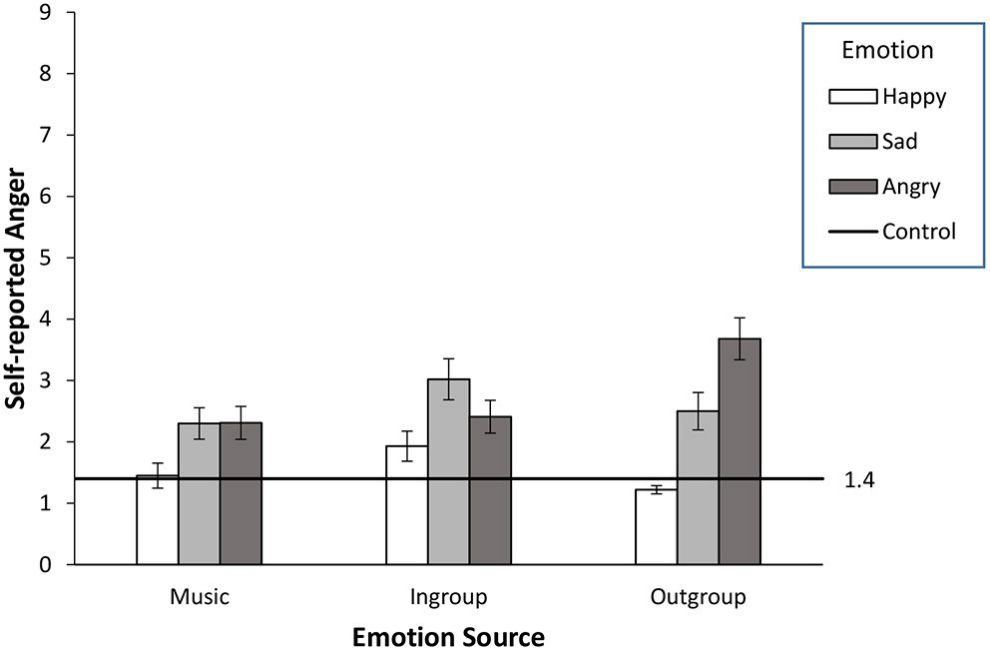
Emotion manipulation checks for Experiment 2 on self-reported anger, as a function of manipulated emotion and emotion source (see main text for simpler patterns detected on self-reported happiness and sadness; *N* = 492 White Male Americans).

Self-reported anger was relatively low on a 0-9 scale and affected by the manipulations in more complex ways, confirming the psychological significance of anger in interethnic settings (see Figure 4). A main effect of emotion (angry, *M* = 2.77, *SD* = 2.08; sad, *M* = 2.59, *SD* = 2.11, happy, *M* = 1.53, *SD* = 1.27, *F*_(2, 421)_ = 19.17, *p* < 0.001, $\eta_{p}^{2}$ = 0.083) was qualified by a significant emotion by emotion source interaction, *F*_(4, 421)_ = 4.47, *p* < 0.005, $\eta_{p}^{2}$ = 0.041. The effect of the emotion manipulation was largest and straightforward in the integral/outgroup source condition, *F*_(2, 140)_ = 21.432, *p* < 0.001, $\eta_{p}^{2}$ = 0.234 vs. incidental/music, *F*_(2, 149)_ = 3.58, *p* < 0.05, $\eta_{p}^{2}$ = 0.046; incidental/ingroup, *F*_(2, 132)_ = 3.69, *p* < 0.05, $\eta_{p}^{2}$ = 0.053. Among integral/outgroup participants, self-reported anger was highest among those who visualized feeling angry (*M* = 3.68, *SD* = 2.26), followed by those who visualized feeling sad (*M* = 2.50, *SD* = 2.16), and lastly those who visualized feeling happy (*M* = 1.22, *SD* = 0.47), all *p*s < 0.005. In the two incidental conditions manipulated anger *and* sadness *both* led to higher levels of self-reported anger, relative to manipulated happiness (sad-anger comparisons both *ns*; happy-sad comparisons both significant; happy-angry comparisons significant only in the incidental/music condition). This psychological confusion between sadness and anger in the incidental emotion settings but not in the integral emotion settings was unexpected. It might reflect a greater readiness to associate negativity to anger in interethnic contact due to anger's higher applicability, over other emotions, to interethnic competitive settings (Cottrell and Neuberg, 2005; Dasgupta et al., 2009). From this standpoint, one could expect valence congruent effects of negative incidental applicable emotions to possibly extend to negative non-applicable emotions when this type of confusion arises. Such confusion did not occur in the integral/outgroup condition, presumably because in that context the attributional frame concerning the outgroup member had been fixed (“they made us sad”); feeling anger associated with someone who made us sad is psychologically challenging given the inconsistent profiles of those two emotions (e.g., in terms of approach and avoidance tendencies). On the other hand, feeling anger associated with the outgroup member when another source has caused our sadness might be less psychologically challenging to reconcile.

Overall, participants displayed greater precision in attributions of anger in the integral/outgroup condition, and undifferentiated anger responses to both negative applicable and non-applicable manipulated emotions in the two incidental conditions.

### Emotion Effects on Interethnic Bias and Mediators

**Figure 5:**
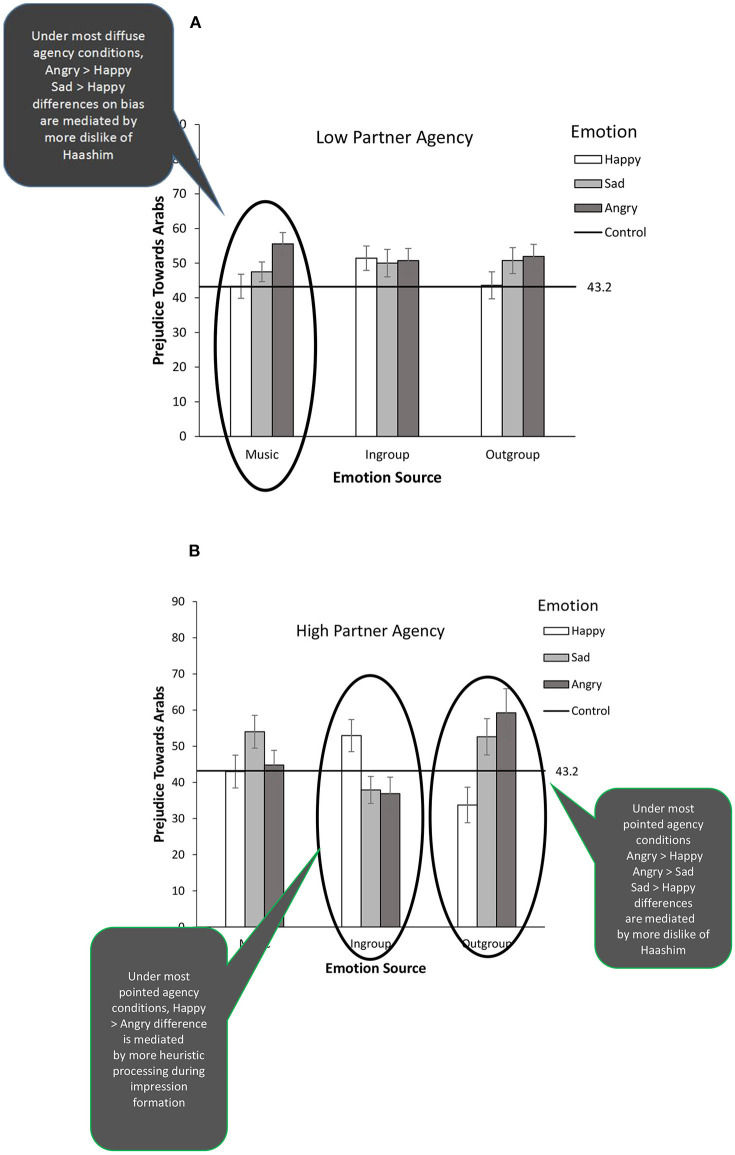
Anti-Arab bias as a function of perceived contact partners' agency, emotion source, and emotion (Experiment 2; *N* = 492 White male Americans engaged in elaborate intergroup imagery with another White American and an Arab American). Error bars represent standard errors and horizontal line bias by baseline control participants not engaging in the intergroup imagery. Significant effects of manipulated emotion are circled and significant mediational results for these effects summarized in associated word balloons.

A 3 (emotion: happy/sad/angry) × 3 (source: music/ingroup/outgroup) × 2 (contact partner agency: low/high) between-subjects factorial ANOVA was performed on the (group-level) outgroup bias index. As predicted, we detected a significant emotion by source interaction, *F*_(4, 412)_ = 5.50, *p* = 0.001, $\eta_{p}^{2}$ = 0.051, qualified by a higher-order emotion × source × partner agency interaction, *F*_(4, 412)_ = 3.00, *p* = 0.018, $\eta_{p}^{2}$ = 0.028; this three-way interaction is displayed in Figure 5. We decomposed this interaction testing the effect of the emotion factor at the varied source and partner agency combinations.

For significant emotion effects at specific source and partner agency combinations, we then tested whether our four mediators—dislike of Haashim, dislike of Matthew, stereotype activation, and non-partner-centered heuristic processing—explained these effects. For this, we used Hayes' (2017) PROCESS Model 4 (v2.15), which allows for testing individual and simultaneous bootstrapped mediators with a dummy-coded multi-categorical independent variable. Hence, we entered (1) the emotion factor as independent variable (dummy coded to contrast its three levels: happy, sad, angry); (2) the four mediators in parallel, and (3) ethnic outgroup bias as dependent variable. We did this separately for participants in the three source-agency condition combinations returning significant emotion effects. Figure 5's balloons summarize significant mediational results which are described in full below; the mediational results were unchanged when mediators were entered individually.

#### Emotion Effects for Those Perceiving Low Partner Agency

Among participants who perceived low agency in the visualized contact partners, the *only* significant effect of manipulated emotion was in the *incidental/music source condition, F*_(2, 87)_ = 3.48, *p* = 0.035, $\eta_{p}^{2}$ = 0.076; all other *ps* > 0.245. This effect (see far left bars in Figure 5's top panel) reflected anti-Arab bias being higher in the music-angry (*M* = 55.56, *SD* = 17.83) than music-happy condition (*M* = 43.33, *SD* = 19.10), *p* = 0.012, and being marginally higher in the music-angry than music-sad condition (*M* = 47.50, *SD* = 15.57), *p* = 0.066. The interethnic bias displayed by the music-angry participants was significantly higher than the bias expressed by baseline control participants (see Figure 5's horizontal line), *p* = 0.010.

This pattern replicates Dasgupta and DeSteno's results of exacerbated intergroup bias under negative incidental-applicable (vs. non-applicable) emotions. However, it also introduces a novel boundary condition: the effect in our data is conditional to the interethnic contact setting having most diffuse subjective agency—the incidental-music/low partner agency condition, which combines a non-agentic diffuse emotion source with non-agentic contact partners. The Dasgupta-DeSteno effect did not materialize under more pointed agency conditions—i.e., with an agentic incidental source and/or agentic contact partners.

Mediational analysis revealed that this valence-congruent effect under most diffuse subjective agency was significantly mediated by dislike of Haashim, but not the other simultaneous mediators (dislike of Matthew, implicit stereotype activation, and non-partner-centered heuristic processing). Specifically, the difference in interethnic bias detected between the music/angry and music/sad conditions, and the difference between the music/angry and the music/happy conditions were statistically explained by increased dislike of Haashim, *b* = 6.68, 95% CI [0.36, 15.63]; *b* = 8.01, 95% CI [0.92, 18.57], respectively. As these effects emerged in a simultaneous mediation model, they reflect the unique explanatory power of outgroup member-to-group generalization, controlling for the other mediators (including stereotype activation).

Hence, among those who perceived the contact partners as non-agentic, anger induced by irritating background music caused increased anti-Arab bias (*a la* DeSteno and Dasgupta), relative to music-induced happiness or sadness, *because* music-induced anger *caused* increased dislike of Haashim and this increased dislike generalized to the outgroup as a whole. These mediational findings are consistent with our expectations for mediators under diffuse subjective agency illustrated in Figure 3.

Thus, against a background of diffuse agency (i.e., a non-agentic emotion source *and* non-agentic contact partners), negative incidental-applicable emotions behaved like negative integral emotions causing valence-congruent effects on interethnic bias, *through* misattribution of emotions to the outgroup (but not ingroup) contact partner, which in turn led to a significant exacerbation of outgroup-level prejudice.

#### Emotion Effects for Those Perceiving High Partner Agency

Among participants who perceived higher agency in the visualized contact partners, the effect of manipulated emotion was significant in the *incidental/ingroup, F*_(2, 54)_ = 4.26, *p* = 0.019, $\eta_{\text{p}}^{2}$ = 0.136, and the *integral/outgroup* conditions, *F*_(1, 57)_ = 5.92, *p* = 0.005, $\eta_{p}^{2}$ = 0.172, but not in the incidental/music condition, *p* = 0.127.

The effect of manipulated emotion detected in the high agency/incidental ingroup condition (see central bars in Figure 5's bottom panel) reflected higher anti-Arab bias among happy-ingroup (*M* = 52.94, *SD* = 17.59), than sad-ingroup (*M* = 37.92, *SD* = 17.44), *p* = 0.012, or angry-ingroup participants (*M* = 36.88, *SD* = 20.24), *p* = 0.015; the sad-ingroup and angry-ingroup conditions did not differ, *p* = 0.861. Bias in the ingroup-happy condition was also marginally higher than in the baseline control condition, *p* = 0.075.

This valence-incongruent effect replicates the happy-sad incidental pattern we detected in Experiment 1 and the counterintuitive biasing effects of incidental-non-applicable emotions documented in early mood research. Once again, however these results convey a more nuanced message. The counterintuitive valence-incongruent pattern materialized exclusively in an interethnic contact setting with very pointed subjective agency—i.e., combining the agentic ingroup source with agentic contact partners. It did not appear in settings with more diffuse agency (i.e., non-agentic sources and/or non-agentic contact partners).

Mediational analyses found the implication of heuristic processing in these incidental-non-applicable emotions' biasing effects. The difference in anti-Arab bias detected between happy-ingroup and angry-ingroup participants was statistically explained by heuristic processing being more pronounced among happy-ingroup participants, *b* = 3.37, 95% CI [0.01, 11.21], while controlling for the other three mediators. No other difference in bias was explained by heuristic processing or other mediators.

Hence, among those who saw high agency in Haashim and Matthew, those who imagined feeling happy (compared to feeling angry) due to what the ingroup contact partner did or said reported more bias toward Arabs in general *because these individuals had paid less attention* to the contact partners and the task at hand. Therefore, in line with mediational expectations in Figure 3 under pointed subjective agency, incidental happiness triggered by a social, ingroup contact partner exacerbated interethnic bias (i.e., a valence incongruent effect) *by* engaging heuristic processing. The null mediational findings for dislike of Matthew (and similarly for bias against the ingroup) indicate that this emotion effect was *not inter*group in nature.

The effect of manipulated emotion in the integral-outgroup/high partner agency condition (far right bars of Figure 5's bottom panel) reflected higher anti-Arab bias in the angry-outgroup (*M* = 52.94, *SD* = 17.59) and sad-outgroup conditions (*M* = 37.92, *SD* = 17.44), than in the happy-outgroup condition (*M* = 36.88, *SD* = 20.24), both *ps* < 0.02. Although the sad-outgroup and angry-outgroup conditions did not differ, *p* = 0.430, of these two conditions only the angry-outgroup condition significantly differed from the baseline control condition, *p* = *0*.044. There was also a weak tendency for the happy-outgroup condition to differ from baseline control, *p* = 0.11, but in the direction of bias attenuation.

These effects replicate the integral condition effect in Experiment 1, and parallel the valence-congruent effects of integral feelings and emotions documented in the intergroup contact literature. Once again, however, these predicted effects come with some novel boundary conditions: In continuity with Graf et al.'s 2014 moderation findings, the effects of integral emotions on interethnic bias (applicable and non-applicable/positive and negative) were circumscribed to interethnic settings with pointed subjective agency (here combining an agentic emotion source with agentic contact partners).

Dislike of the outgroup contact partner Haashim mediated these effects, while controlling for the other mediators. Among those who perceived Haashim and Matthew as highly agentic, differences in interethnic bias between integral/anger, integral/sadness, and integral/happiness were statistically explained by parallel differences in dislike of Haashim; angry-sad, *b* = 15.72, 95% CI [6.26, 31.21]; angry-happy, *b* = 35.26, 95% CI [20.45, 54.58]; sad-happy, *b* = 19.54, 95% CI [10.70, 33.37]. Hence, anger and sadness attributed to what Haashim did or said caused more anti-Arab bias (relative to integral happiness) among those participants *because* these integrally induced negative emotions *made them dislike Haashim* and this increased bias *generalized* to the outgroup as a whole.

These effects and mediational results are consistent with past intergroup contact research. They confirm the centrality of member-to-group generalizations in these effects (Brown and Hewstone, 2005), especially under conditions in which the contact partners are seen to be agentic in shaping the contact experience for the participants (see also Graf et al., 2014).

Overall, our use of an unconfounded emotion induction method and our careful demarcation of differences in emotion sources' and contact partners' agency paid off when identifying boundary conditions and psychological underpinnings for distinguishable emotion effects on interethnic bias. Consistent with *Prediction 1*, incidental/non-applicable-happiness and incidental/non-applicable-sadness had opposite effects on bias compared to their integral/non-applicable counterparts, but only when the incidental source of emotion was an agentic, social source (the ingroup contact partner) and the contact partners were experienced as being highly agentic. These opposing effects of incidental and integral non-applicable emotions were driven (mediated) by distinct psychological processes: non-partner heuristic processing and member-to-group generalization, respectively.

Consistent with *Prediction 2*, incidental/applicable-anger and integral/applicable-anger were found to have similar valence-congruent effects and both exacerbated interethnic bias, relative to baseline control (as well as their happy counterpart). However, these effects presented at diametrically opposite ends of the subjective agency space. The effect of incidental/applicable anger on bias happened at the most diffuse agency end, combining a non-agentic environmental source (music) with non-agentic contact partners. In contrast, the effect of integral/applicable anger happened at the most pointed end with an agentic social source (the outgroup contact partner) and agentic contact partners. Yet their psychological underpinnings were the same: Both incidental/applicable-anger and integral/applicable-anger exacerbated anti-Arab bias by instigating the process of member-to-group generalization. This process involved a misattribution of emotions among incidental/applicable-anger participants, as for these participants there was no legitimate reason for music-induced emotions to instigate bias.

## General Discussion

“… *there are reasons to predict either that the effects of integral and incidental positive affect will differ or that they will not. The empirical resolution of this issue obviously has important implications for whether positive affect arising from intergroup interactions is a help or a hindrance to stereotype change… there are currently no direct comparisons of the impact of incidental and integral affect*” (Mackie et al., 1996, pp. 390-391).

This research set out to deepen understanding of when and how emotions experienced during intergroup contact influence how people feel toward stigmatized outgroups. We sought to return a theory-driven classification of emotions, and clarify which emotions cause bias attenuation and which cause bias exacerbation, thus addressing conceptual and empirical gaps in the intergroup contact literature (Hayward et al., 2017; Kauff et al., 2017). Our research returns a picture of rather nuanced effects.

Surpassing earlier attempts (Wilder and Shapiro, 1989a,b; Echebarria and Fernandez, 2004), we compared incidental and integral emotions experimentally in single designs. We also contrasted negative emotions applicable and non-applicable to the outgroup in Experiment 2. Importantly, we used the same set of materials and tasks across experimental conditions so that we could identify genuine differences and similarities in emotion-prejudice effects, independent of methodological artifacts (Bodenhausen, 1993; Mackie et al., 1996; Wilder and Simon, 1996).

We believe our work is the first to provide the clear comparisons of incidental/integral emotions' influence on intergroup bias that Mackie et al. (1996) were calling for. This work also advances a nuanced, integrative understanding of emotions' effects in contact which considers two established factors (emotion source and emotion applicability), and a novel one (subjective agency). As a result, the outcomes of emotion-focused contact interventions should be more predictable in the future, although not necessarily easy to design.

### Evidence and Implications of Genuine Incidental-Integral Differences and Similarities

Integrating three distinct and otherwise disconnected lines of research on the impact of either incidental *or* integral emotions in a unitary framework (see Figure 1) led us to advance, experimentally test, and find corroborative (although moderated) evidence for two novel, broad predictions for emotion-prejudice effects during interethnic contact. Across two experiments, we demonstrated that incidental-non-applicable and integral-non-applicable happiness and sadness caused opposite effects on interethnic bias (valence-incongruent vs. valence-congruent effects, respectively) in line with *Prediction 1* and past mood and contact research. These effects occurred under conditions of high subjective agency and via distinct mechanisms (non-partner-centered heuristic processing vs. outgroup member-to-group generalization, respectively).

In Experiment 2, we demonstrated that incidental-applicable and integral-applicable anger caused similar valence-congruent effects, and both exacerbated interethnic bias, in line with *Prediction 2* as well as past intergroup contact research and work by DeSteno et al. (2004) and Dasgupta et al. (2009). This only occurred under conditions of *low* subjective agency and via a shared mechanism (outgroup member-to-group generalization).

Our experimental findings therefore allow us to conclude with some assurance that incidental-applicable and integral-applicable emotions produce valence-congruent emotion effects on intergroup bias, whereas incidental-non-applicable emotions produce valence-incongruent effects, at least under some agency conditions.

Because of our use of single designs in meaningful interethnic contact settings with internal and ecological validity, this work gives us confidence that these incidental-integral differences and similarities—unlike past ones (Wilder and Shapiro, 1989a,b; Echebarria and Fernandez, 2004), and inferred ones (Figure 1)—are genuine and ecologically valid. We recognize that Experiment 2's imagined contact procedure does not fare as well in terms of ecological validity as Experiment 1's use of *in vivo*, face-to-face contact; yet, we feel it still does comparatively well-relative to early and more recent incidental emotion research. Our findings add significant parsimony to our theoretical understanding of emotion-prejudice effects and their mechanisms in intergroup contact. We *reduce* emotion-prejudice effects *down* to two distinguishable effects: (1) valence-congruent effects for emotions that are integral or applicable to the outgroup and (2) valence-incongruent patterns for emotions that are incidental-non-applicable, and explain them in terms of two qualitatively distinct mechanisms—outgroup member-to-group generalizations and non-partner-centered heuristic processing. Our mediational tests of mechanisms of the emotion-prejudice link relied on measured mediators; future research should provide experimental manipulations of these processes for more stringent conclusions about their causal implication (Spencer et al., 2005).

Our results provide direct, experimental and mediational evidence for DeSteno and Dasgupta's original idea that incidental-applicable emotions (e.g., incidental-anger in interethnic settings) are functionally equivalent and *not* psychologically distinguishable from their integral counterpart (Wilder and Shapiro, 1989b). Hence, whether Whites' anger during interethnic interactions comes from anger-inducing music in the background or from an obnoxious ethnic contact partner does not seem to matter; *irrespective of its source*, anger exacerbates interethnic bias.

DeSteno and Dasgupta expected these effects to be mediated by outgroup stereotypes and behaviors. We tested these mediational hypotheses in both experiments, once using a non-obtrusive open-ended measure (Experiment 1; see Supplementary Material) and once using a computerized implicit IAT-type measure (Experiment 2), but found no corroboration for their putative mechanism. Instead, in Experiment 2 we found that the isomorphic biasing effects of incidental-applicable and integral-applicable anger on outgroup bias were statistically mediated by increases in bias against the outgroup contact partner. This mechanism could not emerge in DeSteno and Dasgupta' s original research because, unlike our experiments, their research did not use an intergroup contact paradigm.

DeSteno and Dasgupta's research on emotion applicability focused on negative incidental emotions, but their “applicability' principle may extend to integral emotions. An interaction with an anger-inducing ethnic partner may exacerbate Whites' interethnic bias more than an interaction with a sad-inducing ethnic partner, because the former is more applicable to the outgroup. Experiment 2 tested for moderation by emotion applicability with negative *integral* emotions but returned inconclusive results: Integral/applicable-anger, but not integral/non-applicable-sadness, exacerbated bias relative to baseline control (under high partners' agency); however, we found no significant difference in bias between integral-angry and integral-sad participants.

There are reasons to believe that moderation by emotion applicability with *positive* emotions might be hard to achieve. Growing evidence indicates that positive emotions (and words to express them) are more frequent and significantly less differentiated than negative emotions (Bartholow et al., 2001; Rozin and Royzman, 2001). They are more often experienced as default, basic states associated with stability (Rozin and Royzman, 2001; see also Baumeister et al., 2001). As a result, positive emotions might be less amenable than negative emotions to nuanced appraisals (Ellsworth and Scherer, 2003), but this should be established directly.

Our theoretical analysis, paired with fresh unconfounded tests and mediational evidence, suggest singling out *incidental-non-applicable* emotions as emotions that distinctively cause valence *in*congruent effects on intergroup bias via their effects on depth of processing (although our mediation test in Expt. 1 was null, but we attribute that to measure insensitivity). We thus offer closure to historically troubling observations about the impact of incidental emotions: Mood researchers have puzzled over the paradoxical implications of valence-incongruent effects for intergroup relations and bias reduction interventions (Bodenhausen et al., 1994a; Wilder and Simon, 1996). We now confidently classify incidental happiness and sadness as emotions *non-applicable* to ethnic outgroups and to competitive intergroup relations. Similarly, scholars have lamented complexities in the effects of different negative incidental emotions (e.g., incidental sadness and anger; Bodenhausen et al., 1994b; Mackie et al., 1996), that now we know (at least partly) reflect meaningful differences in emotion applicability to interethnic outgroups.

From this clearer classificatory platform for emotions in contact, it becomes easier to predict the outcomes of emotion-driven contact experiences. More precise (but not necessarily practical) designing of emotion-based contact interventions should also be possible. Drawing from our findings, one should be able to better capitalize on the benefits of incidental *and* integral emotions, while minimizing their respective pitfalls. Interventions aimed at reducing interethnic bias should strive to combine integral happiness with incidental sadness (e.g., having ethnic outgroup members provide help to individuals who are in a personal crisis?). Tests of such integral-plus-incidental emotions scenarios would clarify the extent to which joint integral-incidental effects follow a simple additive pattern or less obvious multiplicative patterns. For example, negative incidental- non-applicable emotions, like sadness under high partner agency, might boost benign (vs. pernicious) intergroup judgments because, in addition to their direct effects on intergroup judgments, they serve as an affective standard against which integral emotions can be contrasted. Hence, help from an outgroup member might feel sweet*er* (and result in more bias attenuation) when one is in a sad mood. Of course, integral-plus-incidental combinations might also work in reverse, with multiplicative negative effects. A combination of integral anger and incidental happiness (e.g., being insulted by an ethnic outgroup member at the time of an important personal achievement) might sting *more* (and result in greater bias exacerbation), due to the contrast between one's mood and the outgroup member's actions. Research should examine these non-immediately intuitive predictions (see also, Bodenhausen et al., 2001; Lerner and Keltner, 2001) toward the identification of pathways to bias reduction that are at the same time ecologically sound, ethically appropriate, and politically/socially acceptable.

### Subjective Agency Moderates Emotion-Prejudice Effects and Their Underpinnings

Experiment 2's larger design showed that the valence-congruent and valence-incongruent emotion effects in Predictions 1 and 2 are further complexified by variations in the *agency* of emotion source and contact partners. Emotion induction methods employing highly agentic social sources (e.g. others' story-telling) and non-agentic non-social sources (e.g., weather, hormones) have been used interchangeably or intertwined in past incidental emotion research. Our research demonstrates that agency *matters*.

Happiness stemming from a social incidental source (the other ingroup member in the contact situation) produced bias exacerbation in the context of agentic contact partners. But happiness generated by background music under the same partner agency conditions did not. Anger induced by music against a background of non-agentic contact partners exacerbated bias. But anger stemming from the ingroup contact partner under the same partner agency conditions did not.

Overall, we detected valence-incongruent effects of incidental emotions under pointed agency, but valence congruent effects of incidental emotions under diffuse agency. As anticipated from emotion appraisal theory and attribution theory (Figure 3), our mediational analyses confirmed that emotion source agency and contact partner agency together influenced the psychological underpinnings of these effects. Valence-incongruent effects of incidental-non-applicable emotions under pointed subjective agency were driven by (non-partner-centered) heuristic processing. But valence-congruent effects of incidental-applicable emotions under diffuse subjective agency were driven by (partner-centered) outgroup member-to-group generalizations.

The name given to agency appraisals varies between theories (agency, Roseman, 1984; self-other intent, Frijda, 1986; emotion cause/agent, Scherer, 1994; human agency, Smith and Ellsworth, 1985; see **Table 29**.1 in Ellsworth and Scherer, 2003). Yet, emotion appraisal theories agree that the elicitation and differentiation of emotions depends on people's complex, nuanced and dynamic evaluation of the emotion's circumstances. This includes evaluation of two elements.

First, people try to understand “what (or who) caused the emotion”: its “causation', “responsibility' or “agent' (in our terminology, its source). This might be oneself, someone else, or the environment. Second, for social or animate sources, individuals are motivated to determine the “cause' (e.g., intention, chance) of the emotion. *What motive* drove this source to make me feel this way (Weiner, 1982; Ellsworth and Scherer, 2003). Inferences about intention are typically irrelevant for non-social sources (although people occasionally engage with non-social sources as if they carried intentionality: Wang, 2017).

Causal agency attributions have wide consequences because they establish whether the emotion can be influenced or controlled, has power, and is predictable. Hence, these attributions shape appraisals of ability to cope with the emotion—emotional self-efficacy (Scherer, 1994). Because self-efficacy is intimately linked to fight and flight responses during intergroup contact, as well as willingness to engage in more contact (Trawalter et al., 2009), variations in subjective agency during contact have the potential not only to affect intergroup judgments as we showed in our research, but also to affect long-term intergroup behavioral trajectories for individuals and communities.

### Limitations and Future Research

Our research benefits from combining analyses of emotions with meaningful experiences of interethnic contact. Yet we recognize that the results of the *in vivo* Experiment 1 were quite underpowered, although replicated in Experiment 2, and that there is more work to do on ecological validity. Our Experiment 2 (imagined interethnic contact), in particular, provided a quite static view of emotions as one-off events leading to relatively static and singular appraisals. Emotions in real intergroup contact settings are more dynamic, and appraisals might accumulate and evolve over time (Ellsworth and Scherer, 2003, p. 574). Future research on emotion appraisals should consider the complexities associated with these contact dynamics (Kunda et al., 2002), perhaps using *in vivo* methods like in Experiment 1 or exploiting the flexibility and time-sensitivity afforded by experience sampling methodologies and investigating different types of outgroups.

Our measurement of partner agency in Experiment 2 was also quite rudimentary. It relied on only two items for each of the two contact partners (“active,” “makes decisions easily”). Moreover, while it captured perceptions of the ability of the contact partners to act purposefully or following their will, none of the items reflected the belief that the contact partners willingly caused participants' emotions. Future research on the role of agency in intergroup contact should aim to include more psychometrically robust indicators, which gage contact partners' ability to act autonomously and their perceived intentionality in inducing target emotions.

Our focus in this paper has been on the outgroup. However, Experiment 2's outgroup-plus-ingroup visualization allowed us to explore the impact of our emotion manipulations on bias against the *ingroup* contact partner and the ingroup as a whole. The results are suggestive. Our mediational analyses demonstrated that variations in dislike of Matthew (and White people) following manipulations did *not* explain outgroup bias; hence, those effects can be legitimately regarded as outgroup-driven effects. Although we cannot exclude the possibility that this might be partly due to relatively weak *intergroup* appraisals in Experiment 2's rather rarified visualization paradigm; we know that intergroup appraisals, often marked by elevated levels of ingroup identification, characterize documented experiences of intergroup emotions (Mackie et al., 2009).

In Experiment 2, there was also no evidence that participants misattributed emotions stemming from background music or the outgroup contact partner to the ingroup contact partner and thus the ingroup. Bias against Matthew increased when Matthew caused anger or sadness; but neither these changes nor other factors in our study generalized to bias against the ingroup.

The data therefore suggest ingroup-outgroup asymmetries in emotion-prejudice inferences during contact. People appear more likely to rely on (and make attributional errors concerning) the emotions they experience during contact when inferring how they feel about the outgroup (vs. the ingroup). These asymmetries might reflect differential familiarity and thus schematic complexity of ingroup-outgroup representations, individuals' motivation to protect the ingroup (vs. the outgroup) standing and associated self-esteem, or simply differences in the group salience of outgroup vs. ingroup members. This is worth future investigation.

### Concluding Remarks

This research provides an internally valid and ecologically sound analysis of the interplay between emotion source, emotion applicability, and subjective agency in influencing intergroup bias in interethnic contact experiences. It integrates traditionally separate literatures, and provides an organic and nuanced platform for predicting the effects of emotion-driven contact and for designing bias-reduction strategies that capitalize on integral and incidental emotions. The paradigms we develop combine the strengths of basic incidental emotion research and intergroup contact research while minimizing their respective constraints. We show that incidental emotions that are applicable to the outgroup and integral emotions work in concert. Both shape intergroup bias in valence-congruent ways but at opposite ends of the subjective agency spectrum. Incidental emotions that are non-applicable to the outgroup instead produce oppositional, valence-incongruent effects but only when subjective agency is high.

Our research is the first to attend to agency and causation appraisals as moderators of the emotion-prejudice link during contact. In realistic intergroup contact settings, people have multiple possible explanations for their complex and sometimes ambiguous emotional experiences. In deciding whether to rely on emotions to infer how they feel about the outgroup, people consider *both* the emotion source's and the contact partners' ability to influence their experience. This is a significant contribution to our understanding of emotions in contact and we look forward to future research that further explores emotion appraisals, attributional processes, and their impact on intergroup dynamics during contact.

## Data Availability Statement

The raw data supporting the conclusions of this article will be made available by the authors, without undue reservation.

## Ethics Statement

The studies involving human participants were reviewed and approved by Human Ethics Research Committee, the University of Newcastle, Australia. The patients/participants provided their written informed consent to participate in this study.

## Author Contributions

SP, JH, and MR designed the two experiments. SP prepared materials for Experiment 1 and AL for Experiment 2. SP collected Experiment 1 data. SP and AL collected Experiment 2 data. SP, JH, and MR interpreted the findings, with AL and MM contributing to Experiment 2. All authors contributed to some of the manuscript writing.

## Conflict of Interest

The authors declare that the research was conducted in the absence of any commercial or financial relationships that could be construed as a potential conflict of interest.

## Footnotes

^1^In Experiment 1, we tested the mediating role of several potential drivers of valence congruent/incongruent effects of emotions on interethnic bias, including affect infusion/memory priming, stereotype activation and (non-partner-centered) systematic/heuristic processing. Details of these variables' operationalizations are in the Supplementary Material section. These indicators did not explain our findings on interethnic bias. We carried out new tests with modified indicators in Experiment 2.

^2^Twenty-three participants were excluded for their response to the cultural background question (*n* = 19 reported a non-Anglo background, *n* = 4 failed to respond to this question) and 15 were excluded for their responses to a post-experimental feedback questionnaire, which indicated pointed suspicions about links between the emotion induction phase and the impression formation phase (i.e., “the study perhaps tests the effects of the videoclip on the impressions of Sharna?”). The exclusion of these 15 participants did not change our key results. All participants consented to their data inclusion after full debriefing.

^3^Three participants who were told that the videoclip had been assigned to them on a random basis mistakenly recollected their tutor making the assignment and one participant who read that the videoclip had been assigned to them by their tutor mistakenly recollected it being assigned on a random basis. They might have just failed to read carefully enough the target manipulation framing text. Because the *subjective* attribution of the source of emotion is paramount to the effectiveness and validity of our emotion source manipulation and in light of the limited power constrained by the objective course size for the first study, we re-classified these four participants based on their response to the manipulation check (integral vs. incidental, respectively). Our key results remained unchanged with or without these participants.

^4^A Bayesian 2 (emotion) x 2 (source) ANOVA performed in JASP (0.12.2) found substantial evidence for the interaction effect across matched models (BFIncl = 8.53), using default prior settings (*r* scale fixed effects = 50). The interaction held significant (and unchanged in direction), *F*_(1, 85)_ =8.18, *p* = 0.005, η^2^ = 0.09, when accounting for between-class sessions variance through an expanded ANOVA that had class session treated as a random factor, nested within emotion; lab session was non-significant, *p* = 0.429. We did not use pooled error for our simple effects analyses because homogeneity of variance was significantly violated in these tests; Levene's *F*_(3, 89)_ = 3.85, *p* < 0.05.

^5^For simplicity of reporting we include results for canonical factorial ANOVAs. Results were unchanged with hierarchical regression ANOVA using dummy coding for the experimental factors and target agency entered as a continuous variable.

## References

[B1] Alkire S. (2005). Subjective quantitative studies of human agency. Soc. Indic. Res. 74, 217–260. 10.1007/s11205-005-6525-0

[B2] Allport G. W. (1954). The Nature of Prejudice. Cambridge/Reading, MA: Addison-Wesley.

[B3] Aly A. Green L. (2010). Fear, anxiety and the state of terror. Stud. Confl. Terror. 33, 268–281. 10.1080/10576100903555796

[B4] Amodio D. M. Devine P. G. (2006). Stereotyping and evaluation in implicit race bias: evidence for independent constructs and unique effects on behavior. J. Pers. Soc. Psychol. 91, 652–661. 10.1037/0022-3514.91.4.652 17014291

[B5] Anderson C. A. Anderson K. B. Dorr N. DeNeve K. M. Flanagan M. (2000). Temperature and aggression, in Advances in Experimental Social Psychology, ed Zanna M. P. (Cambridge, MA: Academic Press), 32, 63–133.

[B6] Aslan A. (2009). Islamophobia in Australia. Glebe: Agora Press.

[B7] Augoustinos M. Ahrens C. Innes J. M. (1994). Stereotypes and prejudice: the Australian experience. Br. J. Soc. Psychol. 33, 125–141. 10.1111/j.2044-8309.1994.tb01014.x

[B8] Bartholow B. D. Fabiani M. Gratton G. Bettencourt B. A. (2001). A psychophysiological examination of cognitive processing of and affective responses to social expectancy violations. Psychol. Sci. 12, 197–204. 10.1111/1467-9280.00336 11437301

[B9] Baumeister R. F. Bratslavsky E. Finkenauer C. Vohs K. D. (2001). Bad is stronger than good. Rev. General Psychol. 5, 323–370. 10.1037/1089-2680.5.4.323

[B10] Bodenhausen G. V. (1993). Emotions, arousal, and stereotypic judgments: a heuristic model of affect and stereotyping, in Affect, Cognition, and Stereotyping: Interactive Processes in Group Perception, eds Mackie D. M. Hamilton D. L. (Cambridge, MA: Academic Press), 13–37.

[B11] Bodenhausen G. V. Kramer G. P. Susser K. (1994a). Happiness and stereotypic thinking in social judgment. J. Pers. Soc. Psychol. 66, 621–632. 10.1037/0022-3514.66.4.621

[B12] Bodenhausen G. V. Sheppard L. A. Kramer G. P. (1994b). Negative affect and social judgment: the differential impact of anger and sadness. Eur. J. Soc. Psychol. 24, 445–462. 10.1002/ejsp.2420240104

[B13] Bodenhausen G. V. Mussweiler T. Gabriel S. Moreno K. N. (2001). Affective influences on stereotyping and intergroup relations, in Handbook of Affect and Social Cognition, ed Forgas J. P. (Mahwah, NJ: Lawrence Erlbaum Associates Publishers), 319–343.

[B14] Bower G. H. (1991). Mood congruity of social judgments, in Emotion and Social Judgments, ed Forgas J. P. (Oxford, UK: Pergamon Press), 31–53. 10.4324/9781003058731-3

[B15] Brown R. Hewstone M. (2005). An integrative theory of intergroup contact. Adv. Exp. Soc. Psychol. 37, 255–343. 10.1016/S0065-2601(05)37005-5

[B16] Cafri G. Kromrey J. D. Brannick M. T. (2010). A meta-meta-analysis: empirical review of statistical power, type I error rates, effect sizes, and model selection of meta-analyses published in psychology. Multivariate Behav. Res. 45, 239–270. 10.1080/00273171003680187 26760285

[B17] Conley T. D. Rabinowitz J. L. Rabow J. (2010). Gordon Gekkos, frat boys and nice guys: the content, dimensions, and structural determinants of multiple ethnic minority groups' stereotypes about White men. Anal. Soc. Issues Public Policy 10, 69–96. 10.1111/j.1530-2415.2010.01209.x

[B18] Cottrell C. A. Neuberg S. L. (2005). Different emotional reactions to different groups: a sociofunctional threat-based approach to “prejudice.” J. Personality Soc. Psychol. 88, 770–789. 10.1037/0022-3514.88.5.770 15898874

[B19] Dasgupta N. DeSteno D. Williams L. A. Hunsinger M. (2009). Fanning the flames of prejudice: the influence of specific incidental emotions on implicit prejudice. Emotion 9, 585–591. 10.1037/a0015961 19653784

[B20] DeSteno D. Dasgupta N. Bartlett M. Y. Cajdric A. (2004). Prejudice from thin air: the effect of emotion on automatic intergroup attitudes. Psychol. Sci. 15, 319–324. 10.1111/j.0956-7976.2004.00676.x 15102141

[B21] Diener E. Diener C. (1996). Most people are happy. Psychol. Sci. 7, 181–185. 10.1111/j.1467-9280.1996.tb00354.x

[B22] Dollard J. Miller N. E. Doob L. Mowrer O. H. Sears R. R. (1939). Frustration and Aggression. London, UK: Yale University Press. 10.1037/10022-000

[B23] Echebarria E. A. Fernandez G. E. (2004). The influence of incidental and integral affects on intergroup perception. Revue Int. Psychol. Soc. 17, 51–69.

[B24] Ellsworth P. C. Scherer K. R. (2003). Appraisal processes in emotion, in Handbook of Affective Sciences, eds Davidson R. Scherer K. R Goldsmith H. H. (Oxford, UK: Oxford University Press), 572–595.

[B25] Esses V. M. Zanna M. P. (1995). Mood and the expression of ethnic stereotypes. J. Pers. Soc. Psychol. 69, 1052–1068. 10.1037/0022-3514.69.6.1052

[B26] Faul F. Erdfelder E. Lang A. G. Buchner A. (2007). G^*^Power 3: a flexible statistical power analysis program for the social, behavioral, and biomedical sciences. Behav. Res. Methods 39, 175–191. 10.3758/BF03193146 17695343

[B27] Fiske A. P. (2002). Using individualism and collectivism to compare cultures–A critique of the validity and measurement of the constructs: Comment on Oyserman et al. (2002). Psychol. Bull. 128, 78–88. 10.1037/0033-2909.128.1.78 11843549

[B28] Forgas J. P. (1989). Mood effects on decision making strategies. Aust. J. Psychol. 41, 197–214. 10.1080/00049538908260083

[B29] Forgas J. P. (1994). Sad and guilty? Affective influences on the explanation of conflict in close relationships. J. Pers. Soc. Psychol. 66, 56–68. 10.1037/0022-3514.66.1.56

[B30] Frijda N. H. (1986). The Emotions. Cambridge University Press.

[B31] Fuochi G Voci A. Boin J. Hewstone M. (2020). Affective generalization from intergroup contact: associations between contact-related and outgroup-related empathy, anxiety, and trust. Group Proc. Intergroup Relations. 136843022093266, 1–19. 10.1177/1368430220932662

[B32] Graf S. Paolini S. Rubin M. (2014). Negative intergroup contact is more influential, but positive intergroup contact is more common: assessing contact prominence and contact prevalence in five Central European countries. Eur. J. Soc. Psychol. 44, 536–547. 10.1002/ejsp.2052

[B33] Greenwald A. G. Nosek B. A. Banaji M. R. (2003). Understanding and using the implicit association test: I. An improved scoring algorithm. J. Personality Soc. Psychol. 85, 197–216. 10.1037/0022-3514.85.2.197 12916565

[B34] Haddock G. Zanna M. P. Esses V. M. (1993). Assessing the structure of prejudicial attitudes: the case of attitudes toward homosexuals. J. Pers. Soc. Psychol. 65, 1105–1118. 10.1037/0022-3514.65.6.1105

[B35] Hayward L. E. Tropp L. R. Hornsey M. J. Barlow K. B. (2017). Toward a comprehensive understanding of intergroup contact: descriptions and mediators of positive and negative contact among majority and minority groups. Pers. Soc. Psychol. Bull. 43, 347–364. 10.1177/0146167216685291 28903695

[B36] Hodson G. Costello K. (2007). Interpersonal disgust, ideological orientations, and dehumanization as predictors of intergroup attitudes. Psychol. Sci. 18, 691–698. 10.1111/j.1467-9280.2007.01962.x 17680940

[B37] Huntsinger J. R. Sinclair S. Dunn E. Clore G. L. (2010). Affective regulation of stereotype activation: it's the (accessible) thought that counts. Pers. Soc. Psychol. Bull. 36, 564–577. 10.1177/0146167210363404 20363909 PMC2852264

[B38] Husnu S. Crisp R. J. (2010). Elaboration enhances the imagined contact effect. J. Exp. Soc. Psychol. 46, 943–950. 10.1016/j.jesp.2010.05.014

[B39] Isen A. M. Daubman K. A. (1984). The influence of affect on categorization. J. Pers. Soc. Psychol. 47, 1206–1217. 10.1037/0022-3514.47.6.1206

[B40] Judd C. M. Westfall J. Kenny D. A. (2012). Treating stimuli as a random factor in social psychology: a new and comprehensive solution to a pervasive but largely ignored problem. J. Pers. Soc. Psychol. 103, 54–69. 10.1037/a0028347 22612667

[B41] Kauff M. Asbrock F. Wagner U. Pettigrew T. F. Hewstone M. Schäfer S. J.. (2017). (Bad) feelings about meeting them? Episodic and chronic intergroup emotions associated with positive and negative intergroup contact as predictors of intergroup behavior. Front. Psychol. 8:1449. 10.3389/fpsyg.2017.01449 28900403 PMC5581834

[B42] Kunda Z. Davies P. G. Adams B. D. Spencer S. J. (2002). The dynamic time course of stereotype activation: activation, dissipation, and resurrection. J. Pers. Soc. Psychol. 82, 283–299. 10.1037/0022-3514.82.3.283 11902617

[B43] Lerner J. S. Keltner D. (2001). Fear, anger, and risk. J. Pers. Soc. Psychol. 81, 146–159. 10.1037/0022-3514.81.1.146 11474720

[B44] Leyens J.-P. Yzerbyt V. Y. Schadron G. (1992). The social judgeability approach to stereotypes, in European Review of Social Psychology, eds Stroebe W. Hewstone M. (Hoboken, NJ: Wiley), 3, 91–120. 10.1080/14792779243000032

[B45] Mackie D. M. Maitner A. T. Smith E. R. (2009). Intergroup emotions theory, in Handbook of Prejudice, Stereotyping, and Discrimination, ed Nelson T. D. (London: Psychology Press), 285–307.

[B46] Mackie D. M. Queller S. Stroessner S. J. Hamilton D. L. (1996). Making stereotypes better or worse: multiple roles for positive affect in group impressions, in Handbook of Motivation and Cognition: The Interpersonal Context, eds Sorrentino R. M. Higgins E. T. (New York, NY: Guilford Press), 3, 371–396.

[B47] Macrae C. N. Alnwick K. A. Milne A. B. Schloerscheidt A. M. (2002). Person perception across the menstrual cycle: hormonal influences on social-cognitive functioning. Psychol. Sci. 13, 532–536. 10.1111/1467-9280.00493 12430837

[B48] McDonald M. Navarrete C. D. van Vugt M. (2012). Evolution and the psychology of intergroup conflict: the “warrior male” hypothesis. Philos. Trans. R. Soc. B 367, 670–679. 10.1098/rstb.2011.0301 22271783 PMC3260849

[B49] Miles E. Crisp R. J. (2014). A meta-analytic test of the imagined contact hypothesis. Group Proc. Intergroup Relations 17, 3–26. 10.1177/1368430213510573

[B50] Moors A. Ellsworth P. C. Scherer K. R. Frijda N. H. (2013). Appraisal theories of emotion: state of the art and future development. Emotion Rev. 5, 119–124. 10.1177/1754073912468165

[B51] Osgood C. E. Suci G. Tannenbaum P. (1957). The Measurement of Meaning. Champaign, IL: University of Illinois Press.

[B52] Oswald D. L. (2005). Understanding anti-Arab reactions post-9/11: the role of threats, social categories, and personal ideologies. J. Appl. Soc. Psychol. 35, 1775–1799. 10.1111/j.1559-1816.2005.tb02195.x

[B53] Paolacci G. Chandler J. Ipeirotis P. G. (2010). Running experiments on Amazon Mechanical Turk. Judgment Decision Making 5, 411–419.

[B54] Paolini S. Harris N. Griffin A. S. (2016). Learning anxiety in interactions with the outgroup: towards a learning model of anxiety and stress in intergroup contact. Group Proc. Intergroup Relations 19, 275–313. 10.1177/1368430215572265

[B55] Paolini S. Harwood J. Rubin M. (2010). Negative intergroup contact makes group memberships salient: explaining why intergroup conflict endures. Pers. Soc. Psychol. Bull. 36, 1723–1738. 10.1177/0146167210388667 21051766

[B56] Paolini S. Hewstone M. Voci A. Harwood J. Cairns E. (2006). Intergroup contact and the promotion of intergroup harmony: the influence of intergroup emotions, in Social Identities: Motivational, Emotional, and Cultural Influences, ed Brown R. Capozza D. (London: Psychology Press), 209–238. 10.4324/9780203002971-11

[B57] Paolini S. McIntyre K. (2019). Bad is stronger than good for stigmatized, but not admired outgroups: meta-analytical tests of intergroup valence asymmetry in individual-to-group generalization experiments. Pers. Soc. Psychol. Rev. 23, 3–47. 10.1177/1088868317753504 29473444

[B58] Park J. Felix K. Lee G. (2007). Implicit attitudes toward Arab-Muslims and the moderating effects of social information. Basic Appl. Soc. Psych. 29, 35–45. 10.1080/01973530701330942

[B59] Pettigrew T. F. Tropp L. R. (2008). How does intergroup contact reduce prejudice? Meta-analytic tests of three mediators. Eur. J. Soc. Psychol. 38, 922–934. 10.1002/ejsp.504

[B60] Pettigrew T. F. Tropp L. R. (2013). When Groups Meet: The Dynamics of Intergroup Contact. London: Psychology Press. 10.4324/9780203826461

[B61] Ranganath K. A. Nosek B. A. (2008). Implicit attitude generalization occurs immediately; explicit attitude generalization takes time. Psychol. Sci. 19, 249–254. 10.1111/j.1467-9280.2008.02076.x 18315797

[B62] Roseman I. J. (1984). Cognitive determinants of emotion: a structural theory, in Review of Personality & Social Psychology, ed Shaver P. (Newbury Park, CA: Sage), 5, 11–36.

[B63] Rozin P. Royzman E. B. (2001). Negativity bias, negativity dominance, and contagion. Pers. Soc. Psychol. Rev. 5, 296–320. 10.1207/S15327957PSPR0504_2

[B64] Rydell R. J. Mackie D. M. Maitner A. T. Claypool H. M. Ryan M. J. Smith E. R. (2008). Arousal, processing, and risk taking: consequences of intergroup anger. Pers. Soc. Psychol. Bull. 34, 1141–1152. 10.1177/0146167208319694 18593869

[B65] Scherer K. R. (1994). Toward a concept of “modal emotions”, in The Nature of Emotion: Fundamental Questions, eds Ekman P. Davidson R. J. (London: Oxford University Press), 25–31.

[B66] Scherer K. R. Moors A. (2019). The emotion process: event appraisal and component differentiation. Annu. Rev. Psychol. 70, 719–745. 10.1146/annurev-psych-122216-011854 30110576

[B67] Schwarz N. Clore G. L. (1983). Mood, misattribution, and judgments of well-being: informative and directive functions of affective states. J. Pers. Soc. Psychol. 45, 513–523. 10.1037/0022-3514.45.3.513

[B68] Seger C. R. Banerji I. Hee Park S. Smith E. R. Mackie D. M. (2016). Specific emotions as mediators of the effect of intergroup contact on prejudice: findings across multiple participant and target groups. Cognition Emotion 31, 923–936. 10.1080/02699931.2016.1182893 27206543

[B69] Shaheen J. G. (2003). Reel bad arabs: how hollywood vilifies a people. Annals Am. Acad. Political Soc. Sci. 588, 171–193. 10.1177/0002716203588001011

[B70] Smith E. R. (1993). Social identity and social emotions: toward new conceptualizations of prejudice, in Affect, Cognition, and Stereotyping: Interactive Processes in Group, eds Mackie D. M. Hamilton D. L. (Cambridge, MA: Academic Press), 297–315. 10.1016/B978-0-08-088579-7.50017-X

[B71] Smith C. A. Ellsworth P. C. (1985). Patterns of cognitive appraisal in emotion. J. Pers. Soc. Psychol. 48, 813–838. 10.1037/0022-3514.48.4.813 3886875

[B72] Soutphommasane T. (2012). Don't Go Back to Where You Came From: Why Multiculturalism Works. Randwick: UNSW Press.

[B73] Spencer S. J. Zanna M. P. Fong G. T. (2005). Establishing a causal chain: why experiments are often more effective than mediational analyses in examining psychological processes. J. Pers. Soc. Psychol. 89, 845–851. 10.1037/0022-3514.89.6.845 16393019

[B74] Stephenson P. (2009). Typologies of security: indigenous and Muslim Australians in the post-9/11 imaginary. J. Australian Stud. 33, 473–488. 10.1080/14443050903308691

[B75] Tamir M. Robinson M. D. (2004). Knowing good from bad: the paradox of neuroticism, negative affect, and evaluative processing. J. Pers. Soc. Psychol. 87, 913–925. 10.1037/0022-3514.87.6.913 15598114

[B76] Trawalter S. Richeson J. A. Shelton J. N. (2009). Predicting behavior during interracial interactions: a stress and coping approach. Pers. Soc. Psychol. Rev. 13, 243–268. 10.1177/1088868309345850 19778939

[B77] Vandello J. A. Bosson J. K. (2013). Hard won and easily lost: a review and synthesis of research on precarious manhood. Psychol. Men Masculinity 14, 101–113. 10.1037/a0029826

[B78] Visintin E. P. Green E. G. Pereira A. Miteva P. (2017). How positive and negative contact relate to attitudes towards Roma: comparing majority and high-status minority perspectives. J. Community Appl. Soc. Psychol. 27, 240–252. 10.1002/casp.2309

[B79] Wang W. (2017). Smartphones as social actors? Social dispositional factors in assessing anthropomorphism. Comp. Hum. Behav. 68, 334–344. 10.1016/j.chb.2016.11.022

[B80] Weiner B. (1982). The emotional consequences of causal attributions, in Affect and Cognition, eds Clark M. S. Fiske S. T. (Mahwah, NJ: Lawrence Erlbaum Associates Publishers), 185–209.

[B81] Wilder D. A. Shapiro P. N. (1989a). Effects of anxiety on impression formation in a group context: an anxiety-assimilation hypothesis. J. Exp. Soc. Psychol. 25, 481–499. 10.1016/0022-1031(89)90002-4

[B82] Wilder D. A. Shapiro P. N. (1989b). Role of competition-induced anxiety in limiting the beneficial impact of positive behavior by an out-group member. J. Pers. Soc. Psychol. 56, 60–69. 10.1037/0022-3514.56.1.60 2926617

[B83] Wilder D. A. Simon A. F. (1996). Incidental and integral affect as triggers of stereotyping, in Handbook of Motivation and Cognition: The Interpersonal Context, eds Sorrentino R. M. Higgins E. T. (New York, NY: Guilford Press), 3, 397–419.

