# Supplementary material for: Emotions in Intergroup Contact: Incidental and Integral Emotions' Effects on Interethnic Bias Are Moderated by Emotion Applicability and Subjective Agency
Source: Front Psychol. 2021 May 28;12:588944. doi: 10.3389/fpsyg.2021.588944 (PMC8193362; doi:10.3389/fpsyg.2021.588944)
Supplement: Supplementary file 1 [file Data_Sheet_1.pdf]

**Supplementary Material**

**Putative Mediating Variables and Ancillary Measures Tested in Experiment 1**

Immediately after the interview with the ethnic tutor in front of the classroom, as part of a 'follow-up questionnaire' for the *first* study, we asked participants to freely describe what significant emotions and thoughts the initial video clip had evoked. We counted the number of words each participant wrote as a measure of *systematic processing* (or task engagement) during emotion induction (Bodenhausen, Kramer, & Susser, 1994). Moreover, we had a judge blind to hypotheses and conditions rating these open-ended descriptions for their overall valence (-2 = *very negative*, + 2 = *very* positive), as an index of *affect infusion / memory priming* (Bower, 1991; Forgas, 1994). Next, we checked that participants attended to the source manipulation (see Manuscript's main text). At this point, participants completed a validated unobtrusive open-ended measure of stereotype activation in self-descriptions (Mendoza-Denton, Ayduk, Mischel, Shoda, & Testa, 2001; see Kuhn & McPartland, 1954). For this, they described themselves using single words or short sentences in response to 12 "I am…" stem sentences. Two independent judges, blind to hypotheses and conditions, coded individual stem sentence completions for explicit reference to ethnicity (e.g., "have a pale skin complexion") or the locally relevant Anglo-nonAnglo distinction (e.g., "have a Scottish background"; see also Paolini, Harwood, & Rubin, 2010). To index the strength of *stereotype activation,* we identified the *first* ethnicity-related response within each participant's response set; we used zero to indicate no reference to ethnicity and higher values to indicate earlier and, thus, stronger stereotype activation (range 0-12). Inter-judge agreement was satisfactory (Krippendorff's alpha = .71) and discrepancies were resolved through discussion. Next, we measured the hedonic quality of the contact experience: Participants completed 27 items measuring overall hedonic quality of their ethnic contact experience (or contact quality, Voci & Hewstone, 2003; e.g., "the interview you just witnessed was… pleasant"), anticipated intergroup anxiety (Stephan & Stephan, 2000; e.g., "if you were in a one-to-one meeting with

your tutor, you would feel …anxious”) and other positive and negative intergroup emotions (Fiske, Cuddy, Glick, & Xu, 2002; e.g., “you would feel … admiring”). All items were rated on a 10-point scale (1 = *not at all*, 10 = *very much*) and subjected to a principal components analysis with Promax rotation (Russell, 2002). This extracted three interpretable and separate factors that we made into three reliable aggregate indices. The first factor conveyed *anticipated anxiety* (21.92% explained variance; 6-items, alpha = .87; higher values more anxiety); the second factor conveyed *admiration-engagement* (18.16% explained variance; 9-items, alpha = .81; higher values more admiration and contact engagement); the third factor conveyed *contact informality* (6.80% variance; 3-items, alpha = .60; higher values more perceived informality). We then asked participants to complete two items measuring perceived *tutor's normative fit* or typicality (e.g., “my tutor is typical of what other non-Anglo people are like in general”; 1 = *not at all*, 10 = *very much*; Voci & Hewstone, 2003), which resulted in a reliable index (alpha = .95, $r$ = .90). Finally, without prior warning, we tested participants’ recall of ethnicity-unrelated information conveyed during the tutor’s interview, using 18 cued-recall items (e.g., “her favorite sport is…”; “the car she drives is…”). Responses were scored for errors (0 = *accurate*, 1 = *inaccurate*; see also Mackie et al., 1996) by a judge who was blind to both hypotheses and conditions and summed into an aggregate *heuristic processing* index (range 0-18).

We tested whether our indices of systematic processing, affect infusion, stereotype activation, and heuristic processing (as well as contact hedonic qualities and normative fit) mediated—together or on their own—the emotion by emotion source interaction detected on interethnic bias. For this, we carried out bootstrapped moderated mediation analysis using Hayes’ (2013) PROCESS Model 14, entering manipulated emotion as IV (codes, -1 = happy, + 1 = sad), the indices for the potential mediators as simultaneous (as well as individual) mediators (continuous), interethnic bias as DV, and manipulated emotion source (codes, -1 =

incidental, +1 = integral) as moderator of the mediator-DV links. No indirect effect reached standard levels of significance. We carried out brand new tests with modified indicators in Expt. 2.

## References


Bodenhausen, G. V., Kramer, G. P., & Susser, K. (1994). Happiness and stereotypic thinking in social judgment. *Journal of Personality and Social Psychology, 66*, 621-632.

Bower, G. H. (1991). Mood congruity of social judgments. In J. P. Forgas (Ed), *Emotion and social judgments* (pp. 31-53). Elmsford, NY: Pergamon Press.

Fiske, S. T, Cuddy, A. J. C., Glick, P., & Xu, J. (2002). A model of (often mixed) stereotype content: Competence and warmth respectively follow from perceived status and competition. *Journal of Personality and Social Psychology, 82,* 878-902.

Forgas, J. P. (1994). The role of emotion in social judgements: An introductory review and an Affect Infusion Model (AIM). *European Journal of Social Psychology, 24*, 1–24.

Kuhn, M. H., & McPartland, T. S. (1954). An empirical investigation of self-attitudes. *American sociological review*, *19*(1), 68-76.

Mendoza-Denton, R., Ayduk, O., Mischel, W., Shoda, Y., & Testa, A. (2001). Person× Situation interactionism in self-encoding (Iam… when…): Implications for affect regulation and social information processing. *Journal of Personality and Social Psychology*, *80*(4), 533-544.

Paolini, S., Harwood, J., & Rubin, M. (2010). Negative intergroup contact makes group memberships salient: Explaining why intergroup conflict endures. *Personality and Social Psychology Bulletin*, *36*(12), 1723-1738.

Russell, D. W. (2002). In search of underlying dimensions: The use (and abuse) of factor

analysis in Personality and Social Psychology Bulletin. *Personality and Social Psychology Bulletin, 28*, 1629-1646.

Stephan, W. G., & Stephan, C. W. (2000). An integrated threat theory of prejudice. In S. Oskam (Ed.), *Reducing prejudice and discrimination: The Claremont symposium on applied social psychology* (pp. 23-45), Mahwah, NJ: Erlbaum.

Voci, A., & Hewstone, M. (2003). Intergroup contact and prejudice towards immigrants in Italy: The mediational role of anxiety and the moderational role of group salience. *Group Processes and Intergroup Relations 6,* 37-54.
